# GBM tumors are heterogeneous in their fatty acid metabolism and modulating fatty acid metabolism sensitizes cancer cells derived from recurring GBM tumors to temozolomide

**DOI:** 10.3389/fonc.2022.988872

**Published:** 2022-09-23

**Authors:** Sweta Parik, Juan Fernández-García, Francesca Lodi, Karen De Vlaminck, Marleen Derweduwe, Steven De Vleeschouwer, Raf Sciot, Wietse Geens, Linqian Weng, Francesca Maria Bosisio, Gabriele Bergers, Johnny Duerinck, Frederick De Smet, Diether Lambrechts, Jo A. Van Ginderachter, Sarah-Maria Fendt

**Affiliations:** ^1^ Laboratory of Cellular Metabolism and Metabolic Regulation, VIB-KU Leuven Center for Cancer Biology, VIB, Leuven, Belgium; ^2^ Laboratory of Cellular Metabolism and Metabolic Regulation, Department of Oncology, KU Leuven and Leuven Cancer Institute (LKI), Leuven, Belgium; ^3^ Laboratory of Cellular and Molecular Immunology, Vrije Universiteit Brussel, Brussels, Belgium; ^4^ Myeloid Cell Immunology Laboratory, VIB Center for Inflammation Research, Brussels, Belgium; ^5^ Laboratory for Translational Genetics, VIB-KU Leuven Center for Cancer Biology, VIB, Leuven, Belgium; ^6^ Laboratory for Translational Genetics, Department of Human Genetics, KU Leuven, Leuven, Belgium; ^7^ Laboratory for Precision Cancer Medicine, Translational Cell and Tissue Research, Department of Imaging & Pathology, KU Leuven, Leuven, Belgium; ^8^ Department of Neurosurgery, University Hospital Leuven, Leuven, Belgium; ^9^ Department of Pathology, University Hospital Leuven, KU Leuven, Leuven, Belgium; ^10^ Department of Neurosurgery, UZ Brussel, Jette, Belgium; ^11^ Laboratory of Tumor Microenvironment and Therapeutic Resistance, VIB-KU Leuven Center for Cancer Biology, VIB, Leuven, Belgium; ^12^ Laboratory of Translational Cell & Tissue Research Department of Pathology, University Hospital Leuven, Leuven, Belgium; ^13^ Department of Neurological Surgery, UCSF Comprehensive Cancer Center, University of California San Francisco (UCSF), San Francisco, CA, United States

**Keywords:** glioblastoma, fatty acid metabolism, lipotoxicity, SCD1, FADS2, tumor heterogeneity

## Abstract

Glioblastoma is a highly lethal grade of astrocytoma with very low median survival. Despite extensive efforts, there is still a lack of alternatives that might improve these prospects. We uncovered that the chemotherapeutic agent temozolomide impinges on fatty acid synthesis and desaturation in newly diagnosed glioblastoma. This response is, however, blunted in recurring glioblastoma from the same patient. Further, we describe that disrupting cellular fatty acid homeostasis in favor of accumulation of saturated fatty acids such as palmitate synergizes with temozolomide treatment. Pharmacological inhibition of SCD and/or FADS2 allows palmitate accumulation and thus greatly augments temozolomide efficacy. This effect was independent of common GBM prognostic factors and was effective against cancer cells from recurring glioblastoma. In summary, we provide evidence that intracellular accumulation of saturated fatty acids in conjunction with temozolomide based chemotherapy induces death in glioblastoma cells derived from patients.

## Introduction

Glioblastoma (GBM) is the most common adult diffuse glioma but unfortunately also one of the most difficult to treat cancer types. Despite an intensive treatment regimen consisting of surgical resection followed by radiotherapy with concomitant and adjuvant temozolomide (1), the median survival ranges between 15-16 months with 5-year survival rate of only ~5% (2, 3). Temozolomide (TMZ) induces GBM cell death *via* introducing DNA methylation on purine bases and its efficacy is heavily influenced by molecular factors such as O-6-Methylguanine-DNA Methyltransferase (MGMT) promoter methylation with patients having higher methylation showing better response. Moreover, resistance to TMZ, particularly in recurring tumors interferes with its efficacy (4).

Several studies have revealed that perhaps the most significant contributor to GBM treatment resistance is the inherent inter- and intra-tumoral heterogeneity (5). Verhaak et al. and Phillips et al. defined the existence of three distinct GBM subtypes: proneural, mesenchymal and classical, each defined by a specific gene signature (6, 7). Moreover, several studies that used a multi-sampling technique have shown that at least two of the TCGA-derived genomic subtypes of GBM can appear within the same tumor (8, 9). Subsequently, single-cell based analyses have been used to demonstrate cellular heterogeneity in GBM (10–12). Current theories postulate that intra-tumoral heterogeneity can arise from clonal evolution, presence of stem cells and microenvironmental interactions (13). The latter also includes metabolic interactions. Several studies have shed light on the altered fatty acid (FA) metabolism of GBM cancer cells (14–17). These tumors display an increased rate of fatty acid synthesis, mostly attributed to an increase in sterol response element binding protein (SREBP) mediated gene expression (18–20). Further, GBM cells have elevated stearoyl coA desaturase (SCD) activity. In preclinical studies, SCD inhibition reduces growth of GBM tumors alone or in combination with chemotherapy (21, 22). Wang et al. demonstrated that fatty acid desaturase 2 (FADS2) inhibition in combination with radiotherapy reduced growth of U87 xenografts in nude mice (23). In addition, inhibition of FADS2 as well as fatty acid elongase 2 (Elovl2), has been demonstrated to curb the growth of glioma stem cells (24). Ferraro et al. recently reported that breast cancer cells metastasizing to the brain upregulate the expression of fatty acid synthetase (FASN), the inhibition of which significantly curbed metastases formation (25). However, the extent of lipid metabolism heterogeneity and its role in disease recurrence is yet to be explored.

In this study, we deconvolute the role of fatty acid metabolism in GBM heterogeneity, treatment and recurrence. Using single cell sequencing data of glioblastoma tissue from patients, we observed that the expression of *FASN*, *SCD* and *FADS2* correlated with distinct cell fates within the cancer cell cluster. Subsequently, we show that recurrent patient-derived glioblastoma cells can be re-sensitized to TMZ either by supplementing the fatty acid palmitate to media or by targeting SCD or FADS2.

## Methods

### Single-cell RNA sequencing data pre-processing, clustering, and cancer sub-cluster analysis

**Figure 1:**
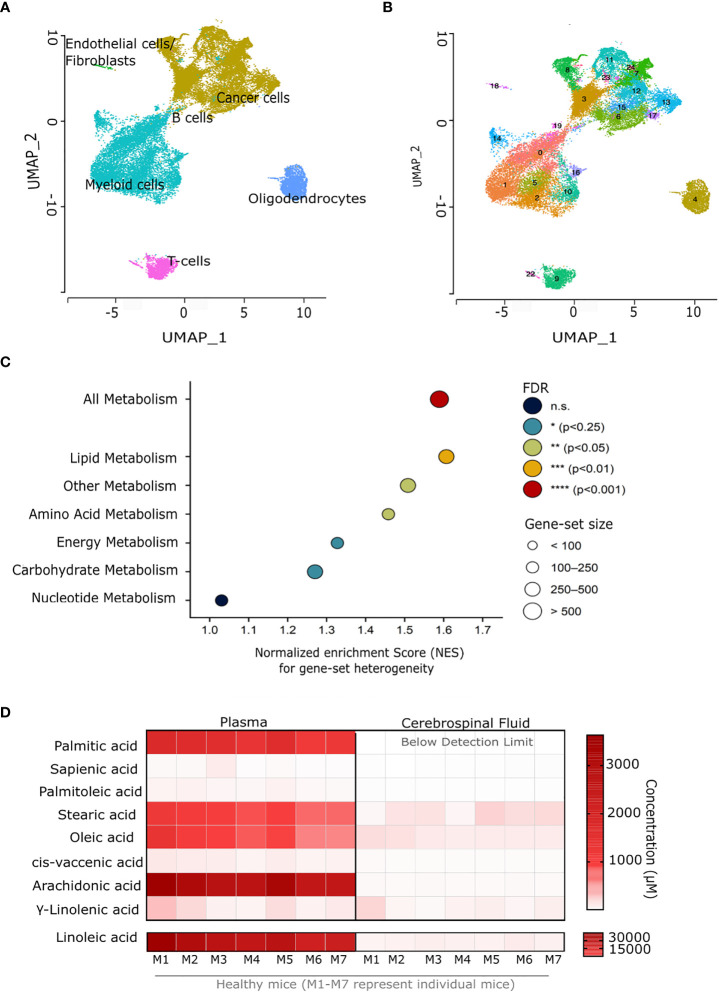
Glioblastoma cancer cells have a highly heterogeneous lipid metabolism. **(A)** Uniform manifold approximation and projection (UMAP) plots of single-cell RNA sequencing data of fresh GBM tissue derived from six patients newly diagnosed with GBM. **(B)** UMAP plots of sub-clustering analysis performed on single-cell RNA sequencing data from fresh GBM tissue derived from six patients newly diagnosed with GBM. **(C)** Results of GSEA-based metabolic pathway-heterogeneity analysis for GBM cancer cells. The y-axis indicates the different KEGG-based gene sets included in the analysis (see Methods), whereas the x axis represents the normalized enrichment scores determined for each gene set based on pre-ranked GSEA. Input genes were ranked based on their standardized variances across all cancer cells in sub-clusters of interest (SC: 6, 7, 8, 11, 17) among those in **Figure 1B**. False discovery rate (FDR)-adjusted p values are indicated by the color scale, with gene-set sizes indicated by the dot areas. **(D)** Total fatty acid concentration (μM) in blood plasma and cerebrospinal fluid from 7 C57BL/6J mice (M1-M7 represent individual mice). n.s., not significant.

After quality control, raw sequencing reads were aligned to the human reference genome GRCh38 and processed to a matrix representing the UMI’s per cell barcode per gene using CellRanger (10x Genomics, v2.0). Raw gene expression matrices generated per sample were merged and analyzed with the Seurat package (v2.3.4). Matrices were filtered by removing cell barcodes with< 401 UMIs,< 200 expressed genes, > 6000 expressed genes or > 20% of reads mapping to mitochondrial RNA. The remaining cells were normalized and genes with a normalized expression between 0.125 and 3, and a quantile-normalized variance > 0.5 were selected as variable genes. When clustering cell types, we regressed out confounding factors: number of UMIs, percentage of mitochondrial RNA, patient ID and cell cycle (S and G2M phase scores calculated by the CellCycleScoring function in Seurat). After regression for confounding factors, all variably-expressed genes were used to construct principal components (PCs) and PCs covering the highest variance in the dataset were selected. The selection of these PCs was based on elbow and Jackstraw plots. Clusters were calculated by the FindClusters function with a resolution between 0.2 and 2 and visualized using the UMAP dimensional reduction method. Differential gene-expression analysis was performed for clusters generated at various resolutions by both the Wilcoxon rank sum test and Model-based Analysis of Single-cell Transcriptomics (MAST) using the FindMarkers function. A specific resolution was selected when known cell types were identified as a cluster at a given resolution, but not at a lower resolution, with the minimal constraint that each cluster has at least 10 significantly differentially expressed genes (FDR < 0.01 with both methods) with at least a 2-fold difference in expression compared to all other clusters. Annotation of the resulting clusters to cell types was based on the expression of marker genes. All major cell types were identified in one clustering step. The cancer cell cluster, comprising a total of 17,649 cells, could be divided into 12 distinct sub-clusters, SC: 3, 6, 7, 8, 11, 12, 13, 15, 17, 21, 23 and 24 (**Figure 1B**). Sub-cluster 21 was removed from subsequent analyses, as it contained only one cell. Sub-clusters 3/12/23/24 were also removed from subsequent analyses, since over 75% of the cells in them were contributed by a single patient, as was sub-cluster 13, for which 85% of the cells were contributed by two patients only (**Supplementary Figure S1A**), rendering the corresponding sub-clusters patient-specific. Out of the remaining sub-clusters, we identified cluster 15 as a potential low-quality cell cluster, based on the substantially lower library size (nUMI, UMI counts) and number of detected genes (nGene) in the cells comprising it, relative to all other sub-clusters (**Supplementary Figures S1B, C**), and therefore removed it too from all subsequent analyses. Consequently, five cancer cell sub-clusters (6, 7, 8, 11, 17) were further analyzed.

### Cancer sub-cluster classification and glioblastoma meta-module score calculation

Cells present in either of the remaining five cancer cell sub-clusters were first examined as a whole to assess the degree of metabolic heterogeneity within the cancer cell population. For this, standardized variances were determined, across all cells in these clusters, for all genes in the data set. This was achieved using Seurat’s FindVariableFeatures function with the “vst” selection method. Genes were then ranked based on their standardized variances, and the resulting ranked gene list was used as input for gene-set enrichment analysis (GSEA). GSEA was performed using the Multilevel method implemented in the R package fgsea (v1.20), with 10,000 permutations and a p-value calculation boundary (eps) of 0. Normalized enrichment scores (NES), representing in this case the degree of heterogeneity for the corresponding gene sets, were determined for a manually assembled collection of seven gene sets. The latter were built based on the KEGG gene-set collection provided in the R package gage (v2.44.0). They consisted of a global metabolism gene set (ALL METABOLISM), comprising all genes present in any of KEGG’s metabolic pathways, as well as five extra gene sets containing all genes present in pathways belonging to either of the following KEGG subcategories: Carbohydrate metabolism, Energy metabolism, Lipid metabolism, Nucleotide metabolism, and Amino acid metabolism. An additional gene set containing all metabolic genes not represented in either of these five subcategories (Other Metabolism) was also included.

The five cancer cell sub-clusters (6, 7, 8, 11, 17) were then classified according to their average gene expression levels of *SCD*, *FADS2* and *FASN*. For this, the raw count matrix was subset to these five sub-clusters, and further normalized to units of counts per 100k reads (CP100k) for each cell, by dividing the UMI counts for every gene in each cell by the total UMI counts for that cell, and then multiplying times 10^5^. Average normalized expression levels for SCD1, FADS2 and FASN for each sub-cluster were then converted into Z-Scores as:


$${\text{Z}}_{\text{g,SC}}=\frac{{\bar{\text{E}}}_{\text{g,SC}}-\text{μ}({\bar{\text{E}}}_{\text{g}})}{\sigma ({\bar{E}}_{g})}$$

where ${\bar{E}}_{g,\text{SC}}$ is the average normalized expression level for gene g in sub-cluster SC , while $\mu ({\bar{E}}_{g})$ and $\sigma ({\bar{E}}_{g})$ are respectively the mean and standard deviation of $\bar{E}g,{\;}_{\text{SC}}$ among all sub-clusters.

Enrichment scores for publicly available gene meta-modules identifying known glioblastoma cell states or fates (5) were determined using the GSVA (gene set variation analysis) package (26). In brief, the raw count matrix was subset to the five sub-clusters of interest and was first subject to size-factor and variance-stabilizing normalization using Monocle. GSVA scores were then determined using default parameters (particularly a Gaussian kernel for cumulative density function estimation, as the input expression matrix is already log-normalized by virtue of the variance stabilization) for a publicly available set of gene meta-modules identifying known glioblastoma cell states or fates (10) (see **Supplementary Table S3**). GSVA scores were then averaged over all cells belonging to every given sub-cluster, and these average GSVA scores were further converted (for plotting purposes) into Z-Scores as:


$${\text{Z}}_{\text{m,SC}}=\frac{{\bar{\text{S}}}_{\text{m,SC}}-\text{μ}({\bar{\text{S}}}_{\text{m}})}{\sigma ({\bar{S}}_{\text{m}})}$$

where ${\bar{\text{S}}}_{\text{m,SC}}$ is the average GSVA score for the gene meta-module m in sub-cluster SC, while $\mu ({\bar{\text{S}}}_{\text{m}})$ and $\sigma ({\bar{\text{S}}}_{\text{m}})$ are respectively the mean and standard deviation of ${\bar{\text{S}}}_{\text{m,SC}}$ among all sub-clusters.

### Cell lines, cell culture and chemicals

Human GBM cells U87 IDH WT, U87 IDH R132H, U251, LN18 and U118 were obtained from ATCC and cultured in Dulbecco’s modified Eagle’s medium (DMEM) with glutamax and pyruvate (Life Technologies) supplemented with 10% heat-inactivated fetal bovine serum (Life Technologies), 1% penicillin (Life Technologies) and 1% streptomycin (Life Technologies). Human adenocarcinoma A549 and human hepatocellular carcinoma HUH7 cells were obtained from ATCC and cultured in DMEM (Life Technologies) supplemented with 10% heat-inactivated fetal bovine serum (Life Technologies), 1% penicillin (Life Technologies) and 1% streptomycin (Life Technologies). Patient-derived GBM cell lines were generated in-house as described below. All cell lines were confirmed to be mycoplasma free, based on routine testing using the MycoAlert Mycoplasma Detection Kit (Lonza).

For MS experiments, low serum conditions (0.5% FBS for A549 and 1% FBS for others) were applied.^13^C_6_-glucose (CLM-1396 Cambridge Isotope Laboratories) was used for labeling experiments. Merck Frosst Cpd 3j (27) was used as an SCD inhibitor. The fatty acids sodium palmitate, stearic acid, palmitoleic acid, oleic acid, cis-vaccenic acid and linolenic acid were purchased from Sigma-Aldrich. Linoleic acid, arachidonic acid, docosahexaenoic acid and γ-linolenic acid were purchased form Cayman chemical. Sapienate (hexadecenoic acid cis-6) was purchased from Matreya LLC. Solvents for metabolite extraction and mass spectrometry were HPLC grade from Sigma-Aldrich.

Temozolomide, 5-azacytidine, 4µ8C (IRE1 inhibitor), GSK2606414 (PERK inhibitor) and aminotriazole were purchased from Sigma-Aldrich. CellEvent™ Caspase-3/7 Green Detection Reagent and Sytox Green were purchased from Life Technologies.

#### Generation of patient-derived GBM cell lines

Patient-derived cell lines were generated either from freshly resected tumors tissue or from frozen dissociated tissue. Patient information is provided in **Supplementary Table S1**.

##### Generation from fresh tumors

Tissue was obtained from patients undergoing surgical resection at UZ Gasthuisberg, Leuven (Belgium) with informed consent (S59804). Tumor tissue was cut into small pieces (1-2mm) and enzymatically dissociated using the MACS Brain Tumor Dissociation Kit (P) (Miltenyi Biotech). Small pieces of tissue were transferred into gentleMACS™ C Tubes (Miltenyi Biotec) containing Buffer X (3890 μl) that was pre-heated at 37°C for 10-15 minutes. As per manufacturer’s instructions, Enzyme N (50μl), Buffer Y (40μl), and Enzyme A (20μl) were added to the C tubes and gently mixed. The gentle MACS programs “h_tumor_02”, “h_tumor_03”, and “m_brain_01” were run, interspersed with incubation at 37°C for 15 and 10 minutes, respectively. The dissociated sample was passed through a 70µM strainer to remove cell clumps and debris. After centrifugation, the pellet was resuspended with 1 ml ACK lysing buffer (Life Technologies) to remove red blood cells and incubated for 1 min at 37°C. The reaction was stopped by adding 8ml of pre-heated NSA medium (Stem Cell Technologies). Cells were then re-centrifuged, aspirated and resuspended in fresh NSA medium. The cells were seeded onto laminin-coated flasks and maintained as described below.

##### Generation from frozen dissociated tumors

Dissociated tumor tissue was obtained from the Center of human genetics, KU Leuven (Belgium) (S61081). Vials with frozen aliquots of dissociated cells were thawed for 2 minutes in a 37°c water bath. Next, the cell suspension was transferred to a 15 ml centrifuge tube containing 9ml pre-heated NSA medium and centrifuged for 5 minutes at 300g. The cell pellets were resuspended in NSA medium, seeded onto laminin-coated flasks and maintained as described below.

### Patient-derived GBM cell lines culture

Patient-derived GBM cell lines were cultured in NSA medium, composed of: reconstituted human NeuroCult™ NS-A Proliferation kit (Stem Cell Technologies) supplemented with 20µg/ml heparin sodium salt (Stem Cell Technologies), 20ng/ml rhEGF (Peprotech), 20ng/ml b-FGF (Peprotech) and 1% antibiotic/antimycotic (Life Technologies). Flasks or plates were coated with 5µg/ml laminin freshly prepared by diluting 1mg/ml laminin solution (Sigma Aldrich) in dPBS. The flasks/plates were then incubated at 37degrees for at least 1 hour. For seeding cells, laminin was aspirated and fresh medium added immediately. For maintenance, cells were scraped off and re-plated into laminin coated flasks. Accutase **(**Life Technologies**)** was used to dissociate neurospheres and to obtain a single cell suspension, when required. All cell lines were confirmed to be mycoplasma free, based on routine testing using the MycoAlert Mycoplasma Detection Kit (Lonza).

### Temozolmide treatment

Patient-derived GBM cells were prepared into a single-cell suspension, seeded in laminin-coated 12-well or 6-well plates (Corning) and grown in a humidified environment at 37°C with 5% CO_2_. After 24 h, the medium was aspirated, cells were washed with dPBS, and replaced with medium containing 250μM TMZ or 0.25% DMSO as control and incubated for 6 days.

### Fatty acid conjugation

10% BSA solution was prepared by dissolving bovine serum albumin in NSA medium (Stem Cell Technologies) without supplements. Next, this solution was incubated in a water bath at 37°C until completely dissolved and then sterile-filtered using a 0.2 micron filter. In the meantime, 100mM stocks of fatty acids were prepared in 50% ethanol (sodium palmitate) or 100% ethanol (all other fatty acids). Subsequently, these were diluted in 10% BSA to obtain 2mM sodium palmitate and 1mM palmitoleate and sapienate. As control, 100% ethanol was diluted 1:50 in 10% BSA and processed the same way as fatty acids. Next, they were incubated in water bath at 37°C for at least 1 hour to allow conjugation. Conjugated fatty acids were aliquoted to avoid freeze-thaw cycles and stored at -20°C.

### ^13^C_6_ glucose labelling experiments

Patient-derived GBM cells were prepared into a single-cell suspension, seeded in laminin-coated 12-well plates (Corning) and grown in a humidified environment at 37°C with 5% CO_2_. After 24 h, the medium was aspirated, cells were washed with dPBS, and replaced with medium containing 10mM 13C6 glucose with/ without 250μM TMZ or 0.25% DMSO as control. Ethanol conjugated with BSA was used as control for fatty acid treatments. Cells were put back in the incubator and incubated for 6 days.

### Cell death analysis

Cell Death analysis was performed using the IncuCyte ZOOM™ Live Cell Analysis System (Essen BioScience, Inc.). Cells were seeded at a standardized density (10k-20k/well) in laminin-coated 96-well flat clear bottom black plates (Corning) in 100μl of culture medium. After 24 hours, 100μl of fresh medium was added containing 2X the final concentration of fatty acids and/or drugs and 1μM of the dead-cell permeant fluorescent dye Sytox Green™ (Final concentration: 0.5μM) (Life Technologies). The plate was then placed in the IncuCyte chamber maintained in a humidified environment at 37°C with 5% CO_2_ and imaged every 2 hours for 9-10 days. Images were captured using the 10X objective in the phase contrast and green fluorescence channel (400ms exposure). Subsequently, phase confluence and number of sytox green positive cells were measured using the IncuCyte ZOOM™ software (Essen BioScience, Inc.). At each time point, the number of sytox green positive cells in a well was normalized to cell confluence in that well to obtain the metric “Cell Death Index”.

### Protein extraction and western blot analysis

Cells were collected in dPBS by scraping and lysed in RIPA lysis and extraction buffer (Thermo Fisher Scientific) supplemented with protease (Merck Sigma) and phosphatase (Merck Sigma) inhibitors. Extracted proteins were quantified using the Pierce BCA Protein Assay Kit (Thermo Fisher Scientific). Subsequently, 40 µg of protein was loaded on a precast gel NuPAGE Novex 4–12% Bis-Tris (Thermo Fisher Scientific). Proteins were then transferred onto a nitrocellulose membrane using an iBlot2 dry blotting system with iBlot2 transfer stacks (Thermo Fisher Scientific). Membranes were incubated for 1 h at room temperature in a blocking solution of 5% BSA in Tris buffer saline 0.05% Tween-20 (TBS-T). Subsequently, the membranes were incubated overnight at 4 °C with primary antibodies against γh2AX (1:2000) (Merck Millipore#05-636) and actin (1:5000) (Sigma-aldrich #A5441). The next day, membranes were incubated with HRP-linked anti-mouse secondary antibody (Cell Signaling Technology, 7076S) used at a dilution of 1:4000 in 5% milk in TBS-T. Bound antibodies were visualized using SuperSignal West Femto Maximum Sensitivity Substrate (Thermo Fisher Scientific). Images were acquired using the ImageQuant LAS 4000 (GE Healthcare).

### RNA isolation and qPCR with reverse transcription

Total RNA was isolated with TRIzol reagent (Life Technologies) and cDNA was prepared using qScript cDNA Synthesis Kit (Quantabio) according to manufacturer’s instructions. qPCR was performed using PerfeCTa SYBR Green SuperMix (Quantabio) on 7500 Fast Real Time PCR System (Applied Biosystems, Life Technologies) or Platinum™ SYBR™ Green qPCR SuperMix-UDG on ViiA 7 Real-Time PCR System (Applied Biosystems, Life Technologies). Amplification was performed at 95 °C for 10 min, followed by 40 cycles of 15 s at 95 °C and 1 min at 60 °C. *RPL19* was used was loading control. Primer sequences of the genes quantified were: *RPL19* Fwd: 5’-ACCCCAATGAGACCAATAAA-3’, *RPL19* Rev: 5’-CGCAAAATCCTCATTCTCCT-3’, *FASN* Fwd: 5’-CGCTCTGGTTCATCTGCTCT-3’, *FASN* Rev: 5’-GAGCGTAGGATGGAATCTCG-3’, *FADS2* Fwd: 5’-GACCACGGCAAGAACTCAAAG-3’, *FADS2* Rev: 5’-GAGGGTAGGAATCCAGCCATT-3’, *SCD* Fwd: 5’-TCTCTGCTACACTTGGGAGC-3’, *SCD* Rev: 5’-GAGCTTTGTAAGAGCGGTGG-3’, *MGMT* Fwd: 5'-CGTTTGCGACTTGGTACTTGG-3', *MGMT* Rev: 5'-CCCTTGCCCAGGAGCTTTAT-3'.

### FACS analysis for activated caspase

Percentage of cells with active caspase was determined using CellEvent™ Caspase-3/7 Green Detection Reagent (Thermo Fischer Scientific) as per manufacturer’s instructions. Briefly, GBM cells were seeded in laminin-coated plates and then treated with temozolomide for 4 days. Next, the medium was aspirated and replaced with fresh medium with/without temozolomide and palmitate for 2 days. Prior to FACS analysis, medium was again aspirated from the cells and supplemented with medium containing 2μM of CellEvent™ Caspase-3/7 Green Detection Reagent. The plate was put in the incubator for 30minutes. Subsequently, the cells were collected by scraping and resuspended in 200 μl of dPBS containing 50μg/ml propidium iodide (PI) for live/dead cell differentiation. Samples were acquired on a FACSCanto™ II analyzer (BD Biosciences) using the FACSDiva™ v8.0 software (BD Biosciences), and further analyzed with FlowJo v10.7 (BD Biosciences). Events were first gated to remove cell debris in the FSC-A vs. SSC-A plane, followed by doublet removal and selection of live (PI-negative) cells. Percentage of Caspase active (FITC +ve) cells were then determined in this population.

### Metabolite extraction from cells, tissue, plasma and CSF

Metabolite extractions were performed as previously described (28, 29). For cell samples, wells were washed with blood bank saline, and cell metabolism was quenched by flash-freezing the plates in liquid nitrogen. For tissues, samples were weighed (5–15 mg) and pulverized (Cryomill, Retsch) under liquid nitrogen conditions. For plasma, 20μl of cold plasma was transferred to an Eppendorf tube. For cerebrospinal fluid, the entire volume that could be obtained from each mouse (5-7μl) was used and the volume noted down for normalization. All samples were stored at –80°C until metabolite extraction. For cells, 800 μl of –20 °C cold 65% methanol was added to the wells and cells were scraped with a pipette tip and suspensions were transferred to Eppendorf tubes. For tissue, plasma or CSF samples, 800 μl of –20 °C cold 65% methanol was directly added to the tube containing pulverized tissue or liquid. Next, 500 μl of –20 °C cold chloroform was added, and samples were vortexed at 4 °C for 10 minutes to extract metabolites. Phase separation was achieved by centrifugation at 4 °C for 10 minutes, after which the chloroform phase (containing the total fatty acid content) was separated and dried by vacuum centrifugation.

### Metabolite measurement *via* GC-MS

Total fatty acid samples were esterified with 500μl 2% sulfuric acid in methanol overnight at 50 °C and extracted by addition of 600μl hexane and 100μl saturated aqueous NaCl. Samples were centrifuged for 5 min and the hexane phase was separated and dried by vacuum centrifugation. Samples were resuspended in hexane, after which total fatty acids were measured with a 7890A GC system (Agilent Technologies) combined with a 5975C or a 7000 inert MSD system (Agilent Technologies). 1μl of each sample was injected in splitless or 3:1 split (7000) mode with an inlet temperature of 270 °C onto a DB35MS column (Agilent Technologies). Helium was used as a carrier gas with a flowrate of 1 ml per min. The oven was held at 80 °C for 1 min and ramped with 5 °C per min to 300 °C. The mass spectrometry system was operated under electron impact ionization at 70 eV and a mass range of 100–650 amu was scanned.

#### Relative quantification of fatty acids

Relative metabolite abundances were determined using an in-house MATLAB script. Total ion counts were normalized to an internal standard (heptadecanoate) and protein content for cell extracts. The protein concentration was quantified using Pierce BCA Protein Assay Kit (Thermo Fisher Scientific). Tissue samples were normalized to tissue weight (in mg).

#### Absolute quantification of fatty acids in plasma and CSF

To quantify the absolute levels of fatty acids, a standard curve was prepared using nine serial dilutions of a fatty acid mix containing palmitate, stearate, sapienate, palmitoleate, cis-vaccenate, oleate, linoleic acid, linolenic acid and γ-linolenic acid. 20μl of each dilution was then extracted and analyzed *via* GC-MS as described above. Metabolite abundances were determined using an in-house MATLAB script. Total fatty acid amount in each sample was calculated by extrapolating it to the standard curve. Concentration (in μM) was then calculated using the known parameters of amount, molecular weight and volume extracted (20μl).

#### Isotopologue distributions

Isotopologue distribution was determined using an in-house MATLAB script. Correction for naturally occurring isotopologues was achieved using Isocor software.

### Collection of mouse blood plasma and cerebrospinal fluid

7-9 weeks old male C57BL/6J mice were purchased from Janvier Labs. Mice were kept in standard housing conditions with 12 h of light and 12 h of dark. Food and water were provided ad libitum. The temperature was kept between 20 and 24 °C and the humidity between 45 and 65%. All mouse experiments were performed according to the regulations of the Ethische Commissie Dierproeven at the Vrije Universiteit Brussel.

#### Blood collection

Blood was withdrawn through heart puncture using EDTA-filled syringes. It was centrifuged at 12,000rcf for 10mins and 20ul of clear plasma was retrieved and instantly frozen.

#### CSF collection

Animals were anesthetized using Ketamine/Xylazine mixture at a dose of 210 mg/kg and 15 mg/kg respectively. Next, the animal was immobilized on its back and 0.5-1 ml of blood collected *via* a blind heart puncture. The animal was then turned on its ventral side and a small subcutaneous incision was made in the back of the head and cisterna magna located by visual inspection. Finally, using a micropipette, the cisterna magna was punctured, making sure to not touch the brain parenchyma or any blood vessels. Due to capillary action, 4-7 µl of CSF will automatically flow into the pipette. The animal was then euthanized according to approved protocols.

### Collection of human glioblastoma samples

Human samples were collected upon ethical approval of local authorities (S65850). Patient information is provided in **Supplementary Table S2**. Tissues were obtained from UZ Brussel, Jette (Belgium) after obtaining informed consent.

### Statistical analysis

Statistical data analysis was performed using GraphPad Prism v.9.3.1 (GraphPad Software) on n ≥ 3 biologically independent replicates. Specific details on statistical tests are presented in the figure legends. All data are presented as mean ± s.d.

### Data availability

**Figure 2:**
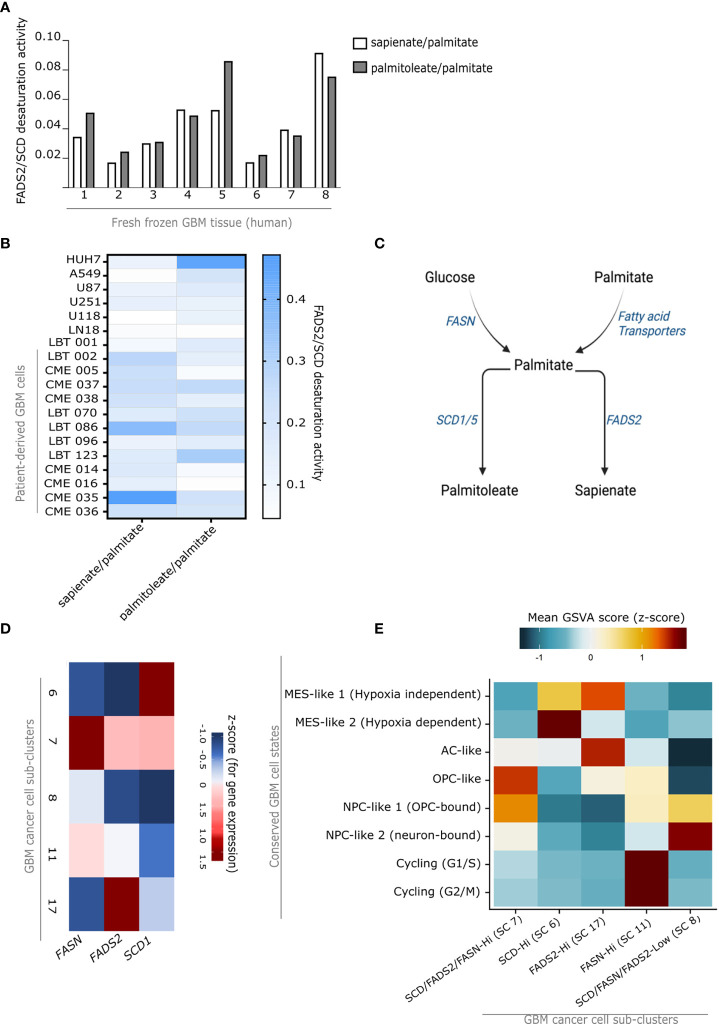
Fatty acid metabolism in GBM cancer cells is linked to cell fate. **(A)** Desaturation activity of SCD (palmitate-to-palmitoleate ratio) and FADS2 (sapienate-to-palmitate ratio) in fresh frozen GBM tissue from eight patients (patient details in **Supplementary Table S2**). **(B)** Desaturation activity of SCD (palmitate-to-palmitoleate ratio) and FADS2 (sapienate-to-palmitate ratio) in human cell lines: hepatocellular carcinoma (HUH7), lung adenocarcinoma (A549), immortalized GBM cells (U87, U251, U118, LN18) and patient-derived GBM cell lines (LBT 001, LBT 002, CME 005, CME 037, CME 038, LBT 070, LBT 086, LBT 096, LBT 123, CME 014, CME 016, CME 035, CME 036) (patient details corresponding to cell lines in **Supplementary Table S1**). Color scale represents mean desaturation activity (n=3). HUH7, U87, U251, LN18, U118 were cultured in 1% FBS medium, A549 cells were cultured in 0.5% FBS medium. Patient-derived GBM cells were cultured in NSA medium without FBS. **(C)** Brief overview of the fatty acid mono-desaturation pathway. **(D)** Average gene expression levels of *FASN*, *SCD1* and *FADS2* in cancer cell sub-clusters of interest (SC: 6, 7, 8, 11, 17) among those in **Figure 1B**. The color scale indicates Z-scores, based on the overall mean expression level (and associated standard deviation) for each gene across all sub-cluster. **(E)** GSVA scores for gene sets representing different GBM cell fates in cancer cell sub-clusters of interest (SC: 6, 7, 8, 11, 17) among those in **Figure 1B**. The color scale indicates Z-scores, based on the overall mean GSVA score (and associated standard deviation) for each gene set across all sub-clusters. MES, mesenchymal; AC, astrocytic cells; OPC, oligodendrocytic precursor cell; NPC, neuronal progenitor cell.

Raw sequencing reads of the human scRNA-seq experiments have been deposited in the controlled access public repository European Genome-phenome Archive (EGA), under study accession number EGAS00001004871. An.xlsx file with each cell/gene set in **Figure 2E** can be found as **Supplementary Table S3**.

## Results

### Glioblastoma cancer cells display a highly heterogeneous lipid metabolism with high sapienate production

To define the intra-tumor heterogeneity of glioblastoma (GBM) tissues, we analyzed the single-cell gene expression profiles of IDH-wt GBM (samples ND1-ND6) from six newly diagnosed patients (30). We identified major cell populations consisting of B cells, T-cells, cancer cells, oligodendrocytes, endothelial cells and fibroblasts using unsupervised clustering (**Figure 1A**). Subsequently, we performed sub-clustering analysis within the cancer cell cluster to better examine intra-tumor heterogeneity. The cancer cell cluster, comprising 17,649 cells, could be divided into 12 distinct sub-clusters (SC: 3, 6, 7, 8, 11, 12, 13, 15, 17, 21, 23 and 24; **Figure 1B**). Next, we selected only cancer cell clusters that were present in most patients, and removed clusters with features indicative of low quality (e.g. low read counts and/or number of genes; see methods and **Supplementary Figure S1**). Based on this, five sub-clusters (SC: 6, 7, 8, 11, 17) were considered for all subsequent analyses. These results highlight the cellular heterogeneity in GBM cancer cells.

Cellular metabolism has been identified as a liability of tumors within the brain (25, 31). Therefore, we further focused on investigating the metabolic heterogeneity of GBM cancer cells. For this, we ranked all genes in the data set based on their degree of variability across all cells in the five cancer cell sub-clusters. This ranked gene list was then used as input for gene set enrichment analysis (GSEA). Normalized enrichment scores (NES) were determined for KEGG-based gene sets representing all metabolic genes, as well as genes involved in specific metabolic pathways (lipid, energy, carbohydrate, amino acid or nucleotide metabolism), and other metabolic genes (i.e. not belonging to any of the latter pathways). Of note, a high NES value indicates here an overrepresentation of the genes present in the corresponding pathway among the most highly-variable genes in the data set, and thus NES values can be interpreted as a proxy for heterogeneity. Our results indicate that GBM cancer cells display an overall high metabolic heterogeneity. We further observed that, within this metabolic heterogeneity, lipid metabolism was the most heterogenous pathway in GBM cancer cells (**Figure 1C**). This suggests that lipid metabolism is a key contributor to the intra-tumor heterogeneity of GBM.

Prior studies have shown that the brain microenvironment is characterized by a deprivation of lipids, and that tumor cells metastasizing to the brain adapt to this lipid-deprived environment by upregulating fatty-acid synthesis (25). In line with these studies, we observed that the cerebrospinal fluid obtained from healthy C57BL/6 mice was severely lipid deprived (**Figure 1D**). This suggests that *de novo* fatty acid synthesis may also be relevant for GBM progression. *De novo* fatty-acid synthesis involves the production of long-chain saturated fatty acids (mainly palmitate) from acetyl-CoA and malonyl-CoA *via* the fatty acid synthetase (FASN), which are then converted into unsaturated fatty acids (e.g. palmitoleate) by desaturase enzymes such as stearoyl-CoA desaturase (SCD) (25). Interestingly, we have previously identified an alternative fatty acid mono-desaturation pathway in cancer, mediated by the delta-6-desaturase FADS2, which produces the unusual fatty acid sapienate from palmitate (28). In line with this, we reported that GBM tumors display very high FADS2 expression (32). This suggests that some cancer cells within GBM tumors may alternatively rely on the desaturation of *de novo* synthesized fatty acids to either palmitoleate, sapienate, or both. Thus, we performed GC-MS based analysis of the total fatty acid in eight human glioblastoma tumors and found that, overall, GBM tumors have nearly equivalent relative SCD activity (palmitoleate to palmitate ratio) and FADS2 activity (sapienate to palmitate ratio) (**Figure 2A**). This indicates that GBM tumors indeed exploit this alternative desaturation pathway and produce considerable amounts of sapienate in addition to palmitoleate. The tumors also displayed significant amounts of arachidonic and docosahexaenoic acid, products of the FADS2 PUFA pathway (**Supplementary Figures S2A, B**). We next compared commercial (U87, U251, U118, LN18) and patient derived GBM cell lines to A549 human lung adenocarcinoma and HUH7 human hepatocellular carcinoma that we have previously characterized (28). We saw that all GBM cell lines, especially those derived from patients had significant sapienate production, comparable to that of the FADS2-high HUH7 cells (**Figure 2B**). Of note, GBM cancer cells were also observed to display a high expression of the above-mentioned fatty acid synthesis and desaturation genes (*FASN, SCD* and *FADS2*) relative to most other cell types in our scRNA-seq data set (**Supplementary Figure S2C**), with the exception of oligodendrocytes and myeloid cells, for which *SCD* was also highly expressed, in agreement with prior reports (33). Taken together, these data suggest a preference for *de novo* fatty acid synthesis and desaturation in GBM cells, a feature that might be attributed to the severe lack of extracellular lipids in the brain microenvironment (**Figure 1D**) (25). They further suggest that GBM cells may rely on either (or both) SCD and FADS2 for fatty-acid desaturation, thus contributing to their heterogeneity at the metabolic level.

We next asked whether the three key genes of *de novo* fatty acid synthesis, namely *FASN*, *FADS2*, and *SCD*, indeed contribute to GBM heterogeneity (**Figure 2C**). Based on the expression of these three genes, the cancer cell clusters could be subdivided into SCD high [SCD-Hi (SC 6)], FADS2 high [FADS2-Hi (SC 17)], FASN high [FASN-Hi (SC 11)], SCD/FADS2/FASN high, [Triple-Hi (SC 7)] and SCD/FADS2/FASN low [Triple-low (SC 8)] cancer cells (**Figure 2D**). We thus next asked whether these different fatty-acid metabolism-defined clusters represented cells with distinct cell fate or function. To address this question, we used gene set variation analysis (GSVA) to determine relative enrichment scores, among all five clusters, for a known set of publicly available gene meta-modules identifying known GBM cell states or fates (10). Remarkably, we found a strong correlation between these expression-annotated sub-clusters and specific cell states. The FASN-Hi sub cluster was strongly correlated with a gene expression module defining cycling cells, whereas SCD-Hi cells displayed a preference for mesenchymal lineage, FADS2-Hi cells displayed an astrocytic lineage signature, Triple-Hi cells displayed a preference for oligodendroglial precursor fate, and Triple-low cells displayed a signature of neural progenitor cells (**Figure 2E**). Thus, these results suggest that the expression levels of lipid metabolism genes are associated with cell fate and potentially functionality in GBM.

Overall, our results demonstrate that GBM cancer cells from patients are metabolically heterogeneous, particularly with regards to lipid metabolism. They further show that the relative expressions of the three key enzymes involved in the fatty acid synthesis pathway is correlated with different cancer cell fates.

### Temozolomide alters fatty acid metabolism in newly diagnosed but not in recurrent glioblastoma

**Figure 3:**
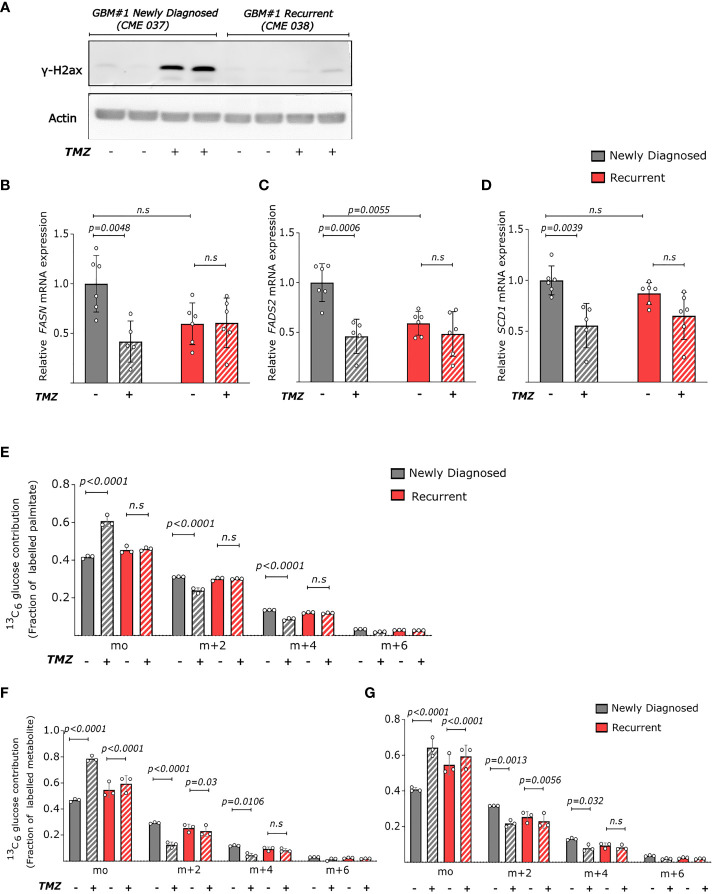
Temozolomide abrogates fatty acid metabolism in newly diagnosed but not recurrent GBM **(A)** Protein levels of phosphorylated h2AX (γh2AX) and actin (loading control) in newly diagnosed (CME 037) and recurrent (CME 038) cells from GBM#1 ± TMZ (250μM). TMZ treatment was done for 6 days. **(B–D)** Relative mRNA levels of **(B)** *FASN*, **(C)** *FADS2* and **(D)** SCD1 *SCD* in newly diagnosed (CME 037) and recurrent (CME 038) cells from GBM#1 ± TMZ (250μM). RPL19 was used as internal control. TMZ treatment was done for 6 days. Statistical analysis was performed using two-way ANOVA with Sidak’s multiple comparison. n = 5-6. **(E-G)** Dynamic labelling of newly diagnosed (CME 037) and recurrent (CME 038) cells from GBM#1 with 13C6-glucose showing ^13^C incorporation into **(E)** palmitate, **(F)** sapienate and **(G)** palmitoleate. TMZ treatment and ^13^C_6_-glucose supplementation was continued for 6 days. Statistical analysis was performed using two-way ANOVA with Tukey’s multiple comparison. n = 3. n.s, not significant.

We next asked whether lipid metabolism contributes to the resistance of reccurring GBM tumors to the chemotherapeutic agent TMZ. We used four pairs of patient-derived cell lines from matched newly diagnosed (ND) and recurrent GBM (RC) tissue of the same patients (patient details in **Supplementary Table S1**). The cells were cultured in low lipid media to resemble the metabolic particularity of the brain (**Figure 1D**). We treated the cell line pairs with TMZ, which induces double stranded break (DSBs) formation in cells, ultimately causing cell death (4). Phosphorylation at ser-139 residue on histone 2A (γh2AX), a marker of DSB formation, was increased in patient derived cell lines from newly diagnosed GBMs cells upon TMZ treatment, while patient derived cell lines from recurrent GBM did not alter h2AX phosphorylation in the presence of TMZ (**Figure 3A**). Thus, in line with the situation in patients, *in vitro* cultured cells derived from recurring GBM had a lower sensitivity to TMZ.

Subsequently, we measured whether TMZ treatment altered the fatty acid metabolism of these cultured GBM cell line pairs. In all four pairs, newly diagnosed GBM showed a significant reduction or a trend towards it in mRNA levels of *FASN, FADS2* and *SCD* (**Figures 3B-D** and **Supplementary Figure S3**) upon TMZ treatment, while recurring GBMs showed either no significant effect or even an increase in the mRNA expression. (**Figures 3B-D** and **Supplementary Figure S3**). To investigate whether these expression changes translated into altered metabolic activities of these enzymes, we performed ^13^C tracing analysis (34) by adding 10mM uniformly labelled glucose (^13^C_6_ glucose) to the culture medium. We observed that the fraction of labelled glucose incorporated into palmitate, sapienate and palmitoleate reduced upon TMZ treatment in cells derived from newly diagnosed GBM#1 but not in recurring GBM#1 (**Figures 3E-G**) while another pair (GBM#4) displayed this effect for palmitate and palmitoleate and not for sapienate (**Supplementary Figures S4A-C**). Overall, these results indicate that chemotherapeutic treatment of GBM cell lines reduces the *de novo* fatty acid synthesis and desaturation, a response that is in part blunted in recurrent GBM cell lines.

### Palmitate supplementation sensitizes GBM to temozolomide

**Figure 4:**
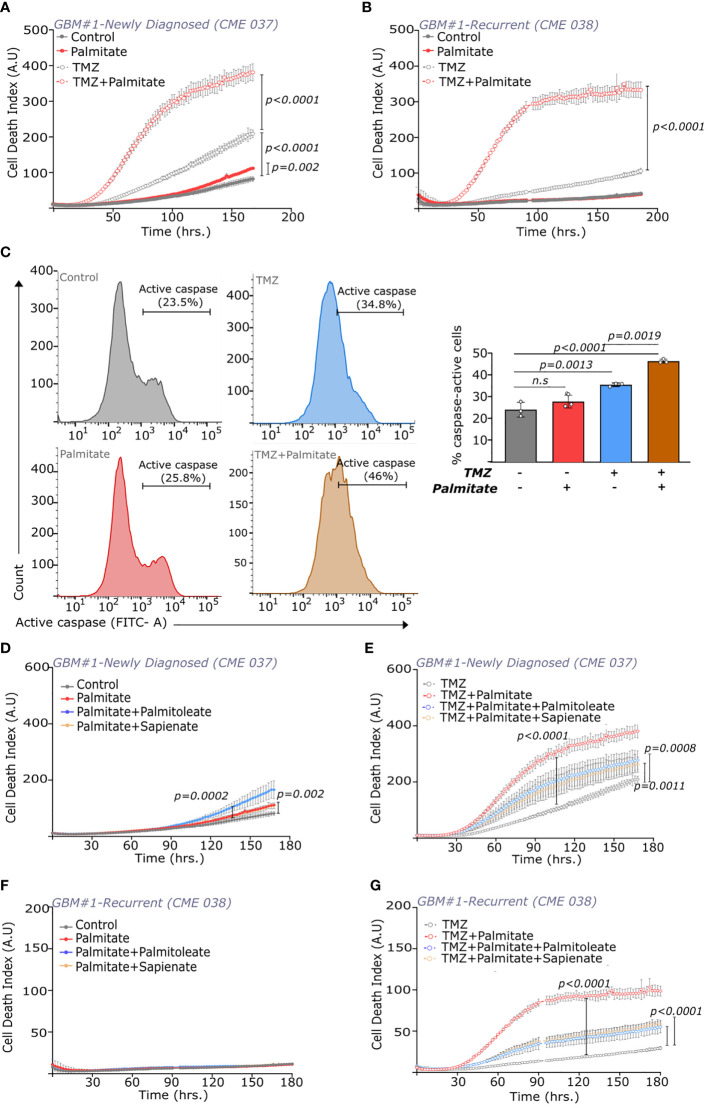
Palmitate influences temozolomide efficacy in newly diagnosed and recurrent GBMs. **(A, B)** Cell death index (number of dead cells/confluence) over time in **(A)** newly diagnosed (CME 037) and **(B)** recurrent (CME 038) cells from GBM#1 with ± TMZ (250μM) and ± palmitate (100μM). Statistical analysis was performed using Two-way ANOVA with repeated measures and Sidak’s multiple compariso n = 5. **(C)** CellEvent™ Caspase-3/7 based FACS analysis of caspase-active population (FITC+ve) in recurring GBM cells (CME 038) from GBM #1 (left) Cell counts vs. FITC intensity in cells with/without TMZ (250 μM) and palmitate (100 μM) (right) Quantitation of the percentage of FITC +ve cells (caspase-active) with/without TMZ (250 μM) and palmitate (100 μM). TMZ treatment was done for 4 days, followed by 2 days of TMZ ± palmitate. Statistical analysis was performed using one-way ANOVA with Tukey’s multiple comparison. n = 3. **(D, E)** Cell death index (number of dead cells/confluence) over time in newly diagnosed (CME 037) cells from GBM#1 **(D)** without or **(E)** with TMZ (250μM) and ± palmitate (100μM), ± sapienate (100μM) or ± palmitoleate (100μM). Statistical analysis was performed using Two-way ANOVA with repeated measures and Sidak’s multiple comparison. n = 5. **(F, G)** Cell death index (number of dead cells/confluence) over time in recurrent (CME 038) cells from GBM#1 **(F)** without or **(G)** with TMZ (250μM) and ± palmitate (100μM), ± sapienate (100μM) or ± palmitoleate (100μM). Statistical analysis was performed using Two-way ANOVA with repeated measures and Sidak’s multiple comparison. n = 5. n.s, not significant.

Saturated and unsaturated lipids need to be balanced to avoid cell death based on lipotoxicity (35). We therefore hypothesized that increasing abundance of palmitate (16:0), a saturated fatty acid, could increase the sensitivity of the GBM cell lines to TMZ. To address this hypothesis, we treated cells with palmitate (100μM) alone or in combination with TMZ (250μM) and monitored cell death overtime using the IncuCyte live-imaging system assay. The cells grew in the presence of sytox green, a fluorescent dye selectively permeant to dead cells (36). We verified that in a suspension of dead and living cells, only dead cells (that are positive for propidium iodide) appear positive for sytox green (**Supplementary Figure S4D**). Cells were imaged every 2 hours for a period of 9-10 days. Subsequently, we measured the number of dead cells, and normalized it to confluence to establish a cell death index. Palmitate alone had a small effect on cell death in cells-derived from newly diagnosed GBM but had no impact on recurring GBM (**Figures 4A, B**). Strikingly, in cell lines derived from both newly diagnosed and recurring GBM, combination of palmitate and TMZ significantly increased cell death compared to the TMZ single treatment in all four pairs (**Figures 4A, B**, **Supplementary Figure S5** and **Supplementary Videos 1-4**). Furthermore, palmitate supplementation with TMZ, increased the percentage of cells with activated caspase as compared to TMZ alone, while palmitate alone had no significant effect (**Figure 4C**). On the other hand, monounsaturated fatty acids, palmitoleate (16:1, n-9) and sapienate (16:1, n-7) had no effect alone or in combination with TMZ (**Supplementary Figures S6, S7**). Further, we also found that this effect was independent of current GBM prognostic factors. Although GBM cells used in this study had highly varied levels of MGMT (**Supplementary Figure S8A**) they all showed drastic cell death induction with palmitate (**Figure 4** and **Supplementary Figure S5**). Also, isogeneic U87 IDH WT and U87 IDH R132H responded similarly to palmitate supplementation (**Supplementary Figures S8B-C**), with the latter displaying hypermethylation. Finally, palmitate was also able to re-sensitize GBM cells treated with the DNA hypomethylating agent 5-azacytidine (37) to TMZ (**Supplementary Figures S8D-E**). Thus, we concluded that palmitate supplementation re-sensitized cells derived from recurring GBM to TMZ.

Induction of cell death based on lipotoxicity is known to be counteracted by the supplementation of unsaturated fatty acids (35). In line, we found that supplementation of the mono-unsaturated fatty acids sapienate and palmitoleate rescued cell death induced by the combination of TMZ with palmitate in GBM cell lines (**Figures 4D-G**). Moreover, modulating the canonical regulators of the cellular lipotoxic response (38) was unable to rescue cell death induced by the combination of TMZ with palmitate. While the PERK inhibitor, GSK2606414 had no effect on palmitate induced lipotoxicity, the IRE1 inhibitor, 4μ8C seemed to further augment the palmitate response (**Supplementary Figure S9**). This suggests that the synergistic action of palmitate with TMZ is not inhibited by targeting conventional ER stress response mechanisms. All together, we found that palmitate supplementation re-sensitized cell lines derived from recurring GBMs to TMZ treatment.

### SCD or FADS2 inhibition enhances temozolomide toxicity in GBM

**Figure 5:**
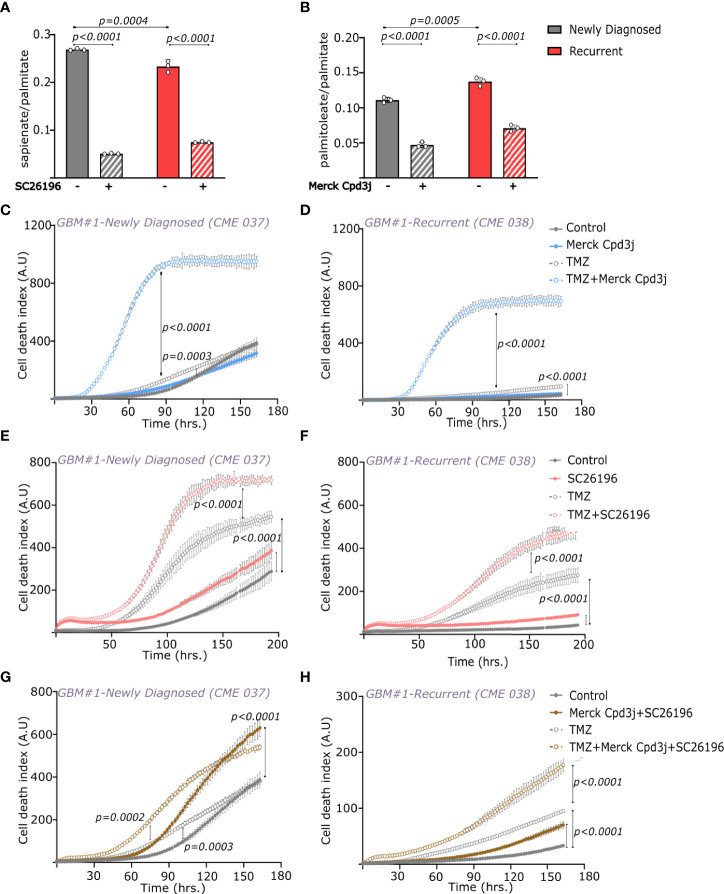
SCD and/or FADS2 inhibition in combination with temozolomide induces cell death in newly diagnosed and recurrent GBMs. **(A)** Sapienate and palmitate balance in newly diagnosed (CME 037) and recurrent (CME 038) cells from GBM#1 with and without FADS2 inhibitor, SC26196 (20μM). Statistical analysis was performed using Two-way ANOVA with Tukey’s multiple comparison. n=3. **(B)** Palmitoleate and palmitate balance in newly diagnosed (CME 037) and recurrent (CME 038) cells from GBM#1 with and without SCD inhibitor, Merck Cpd3j (1nM). Statistical analysis was performed using Two-way ANOVA with Tukey’s multiple comparison. n = 3. **(C, D)** Cell death index (number of dead cells/confluence) over time in **(C)** newly diagnosed (CME 037) and **(D)** recurrent (CME 038) cells from GBM#1 ± TMZ (250μM) and ± SCD inhibitor, Merck Cpd3j (2nM). Statistical analysis was performed using Two-way ANOVA with repeated measures and Sidak’s multiple comparison. n = 5. **(E, F)** Cell death index (number of dead cells/confluence) over time in **(E)** newly diagnosed (CME 037) and **(F)** recurrent (CME 038) cells from GBM#1 ± TMZ (250μM) and ± FADS2 inhibitor, SC26196 (20μM). Statistical analysis was performed using Two-way ANOVA with repeated measures and Sidak’s multiple comparison. n = 5. **(G, H)** Cell death index (number of dead cells/confluence) over time in **(G)** newly diagnosed (CME 037) and **(H)** recurrent (CME 038) cells from GBM#1 ± TMZ (250μM) and ± Merck Cpd3j (2nM)+SC26196 (20μM). Statistical analysis was performed using Two-way ANOVA with repeated measures and Sidak’s multiple comparison. n = 5.

Our observations show that disrupting intracellular FA balance in favor of the saturated fatty acid palmitate has a synergistic effect on TMZ toxicity in both newly diagnosed and recurring GBM cells. Therefore, we next investigated whether pharmacological interventions that accumulate intracellular palmitate may phenocopy the effect of palmitate supplementation. We treated cells with the SCD inhibitor Merck Cpd3j (2nM) or FADS2 inhibitor SC26196 (20µM) either alone or in combination with TMZ. FADS2 and SCD inhibition reduced the sapienate to palmitate and palmitoleate to palmitate ratios respectively, tipping the balance in favor of palmitate (**Figures 5A, B**). The reduction in the sapienate to palmitate ratio was slightly lower in recurrent GBM cells, which is in line with their higher baseline sapienate levels compared to cells from newly diagnosed GBM (**Supplementary Figure S10**). We observed that both newly diagnosed and recurrent GBM cells were slightly sensitive to FADS2 inhibition and insensitive to SCD inhibition (**Figures 5C-F**), which is in line with our finding that glioblastoma cells display high FADS2 mono-desaturation activity leading to sapienate and polyunsaturated fatty acid production. However, when added in combination with TMZ, both SCD and FADS2 inhibition led to a significant induction of cell death (**Figures 5C-F**). Most importantly, this effect was seen in both newly diagnosed and recurrent GBM. Accordingly, supplementation of sapienate (partially) and palmitoleate (fully) rescued TMZ+FADS2 or TMZ+SCD inhibition mediated cell death in newly diagnosed GBM cells, while they both fully rescued cell death in recurring GBM cells (**Supplementary Figure S11**). Combining both SCD and FADS2 inhibition also led to dramatic induction of cell death in both newly diagnosed and recurrent GBM cells (**Figures 5G, H**). Thus, we concluded that both SCD and FADS2 inhibition increased TMZ induced cell death in cell lines derived from not only newly diagnosed but also reccurring GBM tumors.

## Discussion

By combining single cell analysis on patient tissues and *in vitro* techniques, we identified that lipid metabolism plays a crucial role in glioblastoma (GBM). The gene expression levels of *FASN*, *SCD* and *FADS2* were highly heterogeneous in GBM cancer cells and correlated with cell fate. Activities of these key enzyme were reduced upon TMZ treatment but only in newly diagnosed and not recurrent GBM. Perturbing the cellular saturated to monounsaturated fatty acid balance either by extracellular supplementation of palmitate or by inhibition of SCD/FADS2 had a synergistic effect on TMZ treatment. Thus, our work highlights an observation that might be exploited to treat recurrent GBM.

Our study shows that cell fate in glioblastoma tissue is intimately linked with expression of fatty acid synthesis and processing genes, which define distinct sub-clusters within the larger cancer cell population. This is in line with previous findings showing that FASN expression is correlated with stemness (39) and its inhibition induces apoptosis in patient derived GBM cells (40). It was also shown that CD133^+^ glioma stem cells from *in vitro* cultured neurospheres display elevated FADS1 and FADS2 activities (41) and that SCD and FADS2 exhibit spatial heterogeneity with higher expression in the peritumoral area (42).

We find that disrupting the fatty acid balance to accumulate palmitate is very effective at mediating cell death in cultured GBM cells when used in combination with TMZ. While this has not been observed before, the mechanism of lipotoxicity is very consistent with previous work showing that the ratio of monounsaturated to saturated FAs regulates invasiveness and proliferation in melanoma cells (43). In a pancreatic cancer model, increased availability of extracellular palmitate induces lipotoxic cell death and curbs tumor growth while SCD overexpression considerably increases it (35). Lipotoxicity is contrasted by ferroptosis (34, 44), a pathway that is being targeted in several cancers (45, 46). Therein, polyunsaturated fatty acids (PUFAs) incorporated in membranes undergo oxidation and trigger an iron-mediated cell death (47, 48). Shifting fatty acid balance by external supplementation of monounsaturated fatty acids rescues ferroptosis (49) while depleting them *via* SCD or FADS2 inhibition augments it (50, 51).

Disease recurrence remains the biggest challenge in GBM management and there is no standard of care for patients with reccurring GBM (52, 53). Our findings imply that targeting fatty acid desaturation in combination with TMZ to accumulate toxic saturated fatty acids might prove beneficial to these patients. Further, the effect of palmitate accumulation was independent of disease status, DNA methylation status, MGMT levels, IDH status, all of which are crucial GBM prognostic factors and hinder with chemotherapy. Therefore, our data imply that palmitate accumulation might have therapeutic benefits in patients that do not respond to conventional therapy. Although it may seem paradoxical that higher levels of fatty acids might provide a survival advantage, a large number of studies has shown that higher BMI correlates with improved survival in glioblastoma patients (54–57), though this is contested by some other reports (58, 59). Moreover, fatty acid rich ketogenic diet also provides a survival advantage in glioblastoma (60). Yet, it remains to be determined whether this is due to lipid accumulation in cancer cells or systemic effects. Enrichment of saturated fatty acids is not a feasible treatment option as they can have a negative impact on patient health. Therefore, pharmacological means such as SCD and FADS2 inhibition are attractive targets and do extend survival in preclinical studies (21–23, 61). In addition, we have previously reported that a combination of SCD and FADS2 inhibition successfully curbs tumor growth in an orthotopic hepatocellular carcinoma model, albeit not necessarily involving the same mechanism (28). Rudalska et al. reported in HCC that induction of lipotoxicity by targeting SCD1 had a significant effect on tumor growth (62). It would, therefore, be interesting to explore whether this treatment regimen would benefit other cancers besides GBM.

## Data availability statement

The datasets presented in this study can be found in online repositories. The names of the repository/repositories and accession number(s) can be found below: https://ega-archive.org/, EGAS00001004871.

## Ethics statement

The studies involving human participants were reviewed and approved by Ethical Committee KU Leuven, UZ Leuven, VUB and UZ Brussels. The patients/participants provided their written informed consent to participate in this study. The animal study was reviewed and approved by Ethische Commissie Dierproeven at the Vrije Universiteit Brussel.

## Author contributions

S-MF and SP designed the study; SP conducted the experiments and analyzed data; JF-G and FL analyzed the single-cell sequencing data. KDV isolated blood and cerebrospinal fluid from mice. MD, SDV and RS collected and generated patient-derived GBM cells supervised by FDS and FMB. JD performed the surgeries on patients and WG assisted with collection of human GBM tumors. LW assisted with analysis of single-cell sequencing data supervised by GB. SP, S-MF, JF-G, and JVG wrote and edited the manuscript. S-MF and JVG supervised the study. All authors contributed to the article and approved the submitted version.

## Funding

SP received an international PhD scholarship from VIB. JF-G is a FWO postdoctoral fellow. JVG is supported by iBOF, EOS, FWO, Stichting tegen Kanker, Kom op tegen Kanker and EU-Innovative Medicines Initiative research grants. S-MF acknowledges funding from the European Research Council under the ERC Consolidator Grant Agreement n. 771486–MetaRegulation, FWO – Research Projects, KU Leuven – FTBO, King Baudouin Foundation, Beug Foundation, Stichting tegen Kanker and Fonds Baillet Latour.

## Acknowledgments

We thank all patients for their valuable contribution. We thank all members of Fendt lab and Van Ginderachter lab for their feedback.

## Conflict of interest

S-MF has received funding from Bayer AG, Merck and Black Belt Therapeutics, has consulted for Fund+ and is in the advisory board of Alesta Therapeutics. JVG collaborates with Montis and has consulted for Fund+.

The remaining authors declare that the research was conducted in the absence of any commercial or financial relationships that could be construed as a potential conflict of interest.

## Publisher’s note

All claims expressed in this article are solely those of the authors and do not necessarily represent those of their affiliated organizations, or those of the publisher, the editors and the reviewers. Any product that may be evaluated in this article, or claim that may be made by its manufacturer, is not guaranteed or endorsed by the publisher.
