# Supplementary material for: GBM tumors are heterogeneous in their fatty acid metabolism and modulating fatty acid metabolism sensitizes cancer cells derived from recurring GBM tumors to temozolomide
Source: Front Oncol. 2022 Sep 23;12:988872. doi: 10.3389/fonc.2022.988872 (PMC9635944; doi:10.3389/fonc.2022.988872)
Supplement: Supplementary file 1 [file DataSheet_1.zip › Images/Supplementary Image Captions.docx]

Supplementary Material

**Figure legends**

**Supplementary Figure S1: Quality control of GBM single-cell RNA sequencing data.**

1. Percentage of cells from each patient in GBM cell sub-clusters.
2. Log transformed read counts per cell (log_10_ nUMI) in GBM cancer cell sub-clusters.
3. Log transformed no. of detected genes per cell (log_10_ nGene) in GBM cancer cell sub-clusters.

**Supplementary Figure S2: GBM tissues produce FADS2-mediated polyunsaturated fatty acids.**

1. Metabolite levels of arachidonic acid in fresh frozen GBM tissue from 8 patients (patient details in Supplementary Table S2).
2. Metabolite levels of docosahexaenoic acid in fresh frozen GBM tissue from 8 patients (patient details in Supplementary Table S2).
3. Normlized expression of *FADS2, FASN* and *SCD* in B-cells, T-cells, oligodendrocytes, cancer cells, myeloid cells and endolthelial cells/fibroblasts determined via scRNA sequencing.

**Supplementary Figure S3: Temozolomide abrogates fatty acid metabolism in newly diagnosed but not recurrent GBM**

(A-C) Relative mRNA levels of (A) *FASN*, (B) *FADS2* and (C) *SCD* in newly diagnosed (CME 014) and recurrent (CME 016) cells from GBM#2 ±TMZ (250μM). RPL19 was used as internal control. TMZ treatment was done for 6 days. Statistical analysis was performed using two-way ANOVA with Sidak’s multiple comparison. n=3.

(D-F) Relative mRNA levels of (D) *FASN*, (E) *FADS2* and (F) *SCD* in newly diagnosed (CME 035) and recurrent (CME 036) cells from GBM#3 ±TMZ (250μM). RPL19 was used as internal control. TMZ treatment was done for 6 days. Statistical analysis was performed using two-way ANOVA with Sidak’s multiple comparison. n=3.

(G-I) Relative mRNA levels of (G) *FASN*, (H) *FADS2* and (I) *SCD* in newly diagnosed (CME 005) and recurrent (LBT 002) cells from GBM#4 ±TMZ (250μM). RPL19 was used as internal control. TMZ treatment was done for 6 days. Statistical analysis was performed using two-way ANOVA with Sidak’s multiple comparison. n=3.

**Supplementary Figure S4: Temozolomide abrogates *de novo* fatty acid synthesis and desaturation in newly diagnosed but not recurrent GBM.**

(A-C) Dynamic labelling of newly diagnosed (CME 005) and recurrent (LBT 002) cells from GBM#4 with 13C6-glucose showing ^13^C incorporation into (A) palmitate, (B) sapienate and (C) palmitoleate. TMZ treatment and ^13^C_6_-glucose supplementation was continued for 6 days. Statistical analysis was performed using two-way ANOVA with Tukey’s multiple comparison. n = 3.

1. FACS analysis of GBM cells co-stained with propidium iodide (PI) and sytox green. PI-positive and negative cells were gated in the single cells. Sytoz green intensity was then determined in cells within the PI-positive and negative gates.

**Supplementary Figure S5: Palmitate influences temozolomide efficacy in newly diagnosed and recurrent GBMs.**

(A-B) Cell death index (number of dead cells/confluence) over time in (A) newly diagnosed (CME 014) and (B) recurrent (CME 016) cells from GBM#2 with ±TMZ (250μM) and ±palmitate (100μM). Statistical analysis was performed using Two-way ANOVA with repeated measures and Sidak’s multiple comparison. n=5.

(C-D) Cell death index (number of dead cells/confluence) over time in (A) newly diagnosed (CME 035) and (B) recurrent (CME 036) cells from GBM#3 with ±TMZ (250μM) and ±palmitate (100μM). Statistical analysis was performed using Two-way ANOVA with repeated measures and Sidak’s multiple comparison. n=5.

(E-F) Cell death index (number of dead cells/confluence) over time in (A) newly diagnosed (CME 005) and (B) recurrent (LBT 002) cells from GBM#4 with ±TMZ (250μM) and ±palmitate (100μM). Statistical analysis was performed using Two-way ANOVA with repeated measures and Sidak’s multiple comparison. n=5.

**Supplementary Figure S6: Palmitoleate and sapienate supplementation has no effect on temozolomide efficacy in newly diagnosed and recurrent GBMs.**

(A-B) Cell death index (number of dead cells/confluence) over time in newly diagnosed (CME 037) cells from GBM#1 (C) without or (D) with TMZ (250μM) and ± sapienate (50μM) or ±palmitoleate (50μM). Statistical analysis was performed using Two-way ANOVA with repeated measures and Sidak’s multiple comparison. n=5. Control plots same as that in Figure 4.

(C-D) Cell death index (number of dead cells/confluence) over time in recurrent (CME 038) cells from GBM#1 (C) without or (D) with TMZ (250μM) and ± sapienate (50μM) or ±palmitoleate (50μM). Statistical analysis was performed using Two-way ANOVA with repeated measures and Sidak’s multiple comparison. n=5. Control plots same as that in Figure 4.

(E-F) Cell death index (number of dead cells/confluence) over time in newly diagnosed (CME 014) cells from GBM#2 (E) without or (F) with TMZ (250μM) and ± sapienate (50μM) or ±palmitoleate (50μM). Statistical analysis was performed using Two-way ANOVA with repeated measures and Sidak’s multiple comparison. n=5. Control plots same as that in Figure S5.

(G-H) Cell death index (number of dead cells/confluence) over time in recurrent (CME 016) cells from GBM#2 (G) without or (H) with TMZ (250μM) and ± sapienate (50μM) or ±palmitoleate (50μM). Statistical analysis was performed using Two-way ANOVA with repeated measures and Sidak’s multiple comparison. n=5. Control plots same as that in Figure S5.

**Supplementary Figure S7: Palmitoleate and sapienate supplementation has no effect on temozolomide efficacy in newly diagnosed and recurrent GBMs.**

(A-B) Cell death index (number of dead cells/confluence) over time in newly diagnosed (CME 035) cells from GBM#3 (C) without or (D) with TMZ (250μM) and ± sapienate (50μM) or ±palmitoleate (50μM). Statistical analysis was performed using Two-way ANOVA with repeated measures and Sidak’s multiple comparison. n=5. Control plots same as that in Figure S5.

(C-D) Cell death index (number of dead cells/confluence) over time in recurrent (CME 036) cells from GBM#3 (E) without or (F) with TMZ (250μM) and ± sapienate (50μM) or ±palmitoleate (50μM). Statistical analysis was performed using Two-way ANOVA with repeated measures and Sidak’s multiple comparison. n=5. Control plots same as that in Figure S5.

(E-F) Cell death index (number of dead cells/confluence) over time in newly diagnosed (CME 005) cells from GBM#4 (E) without or (F) with TMZ (250μM) and ± sapienate (50μM) or ±palmitoleate (50μM). Statistical analysis was performed using Two-way ANOVA with repeated measures and Sidak’s multiple comparison. n=5. Control plots same as that in Figure S5.

(G-H) Cell death index (number of dead cells/confluence) over time in recurrent (LBT 002) cells from GBM#4 (G) without or (H) with TMZ (250μM) and ± sapienate (50μM) or ±palmitoleate (50μM). Statistical analysis was performed using Two-way ANOVA with repeated measures and Sidak’s multiple comparison. n=5. Control plots same as that in Figure S5.

**Supplementary Figure S8: Palmitate influences temozolomide efficacy independent of common prognostic factors.**

(A) mRNA levels of MGMT in all cell lines. RPL19 was used as internal control.

(B-C) No. of dead cells over time in (A) U87 IDH WT and (B) U87 IDH R132H cells with ±TMZ (250μM) and ±palmitate (100μM). Statistical analysis was performed using Two-way ANOVA with repeated measures and Sidak’s multiple comparison. n=5.

(D-E) No. of dead cells over time in U87 IDH WT cells (A)without and (B) with 5-azacytidine (5μM) and ±TMZ (250μM) or ±palmitate (100μM). Statistical analysis was performed using Two-way ANOVA with repeated measures and Sidak’s multiple comparison. n=5.

**Supplementary Figure S9: IRE1 or PERK pathways do not regulate palmitate mediated lipotoxicity in GBM cells.**

1. Cell death index (number of dead cells/confluence) over time in newly diagnosed (CME 037) cells from GBM#1 with ±TMZ (250μM) and ±palmitate (100μM), PERK inhibitor, GSK2606414 (25nM) and IRE1 inhibitor, 4μ8C (25μM). Statistical analysis was performed using Two-way ANOVA with repeated measures and Sidak’s multiple comparison. n=5.
2. Cell death index (number of dead cells/confluence) over time in recurrent (CME 038) cells from GBM#1 with ±TMZ (250μM) and ±palmitate (100μM), PERK inhibitor, GSK2606414 (25nM) and IRE1 inhibitor, 4μ8C (25μM). Statistical analysis was performed using Two-way ANOVA with repeated measures and Sidak’s multiple comparison. n=5.

**Supplementary Figure S10: Patient-derived GBM cells produce sapienate over arachidonate *in vitro****.*

1. Metabolite levels of sapienate in newly diagnosed (CME 037) and recurrent (CME 038) cells from GBM#1. Statistical analysis was performed using unpaired two-tailed t-test. n=3.
2. Metabolite levels of arachidonate in newly diagnosed (CME 037) and recurrent (CME 038) cells from GBM#1. Statistical analysis was performed using unpaired two-tailed t-test. n=3.

**Supplementary Figure S11: Supplementation of monounsaturated fatty acids rescue cell death induced by FADS2 and SCD inhibition with temozolomide.**

1. Cell death index (number of dead cells/confluence) over time in newly diagnosed (CME 037) cells from GBM#1 ±TMZ (250μM) and ± SCD inhibitor, Merck Cpd3j (2nM) with palmitoleate (50μM) or ± FADS2 inhibitor, SC26196 (20μM) with sapienate (50μM). Statistical analysis was performed using Two-way ANOVA repeated measures and Sidak’s multiple comparison. n=5.
2. Cell death index (number of dead cells/confluence) over time in recurrent (CME 038) cells from GBM#1 ±TMZ (250μM) and ± SCD inhibitor, Merck Cpd3j (2nM) with palmitoleate (50μM) or ± FADS2 inhibitor, SC26196 (20μM) with sapienate (50μM). Statistical analysis was performed using Two-way ANOVA repeated measures and Sidak’s multiple comparison. n=5.
