# Supplementary figures and images for: GBM tumors are heterogeneous in their fatty acid metabolism and modulating fatty acid metabolism sensitizes cancer cells derived from recurring GBM tumors to temozolomide
Source: Front Oncol. 2022 Sep 23;12:988872. doi: 10.3389/fonc.2022.988872 (PMC9635944; doi:10.3389/fonc.2022.988872)

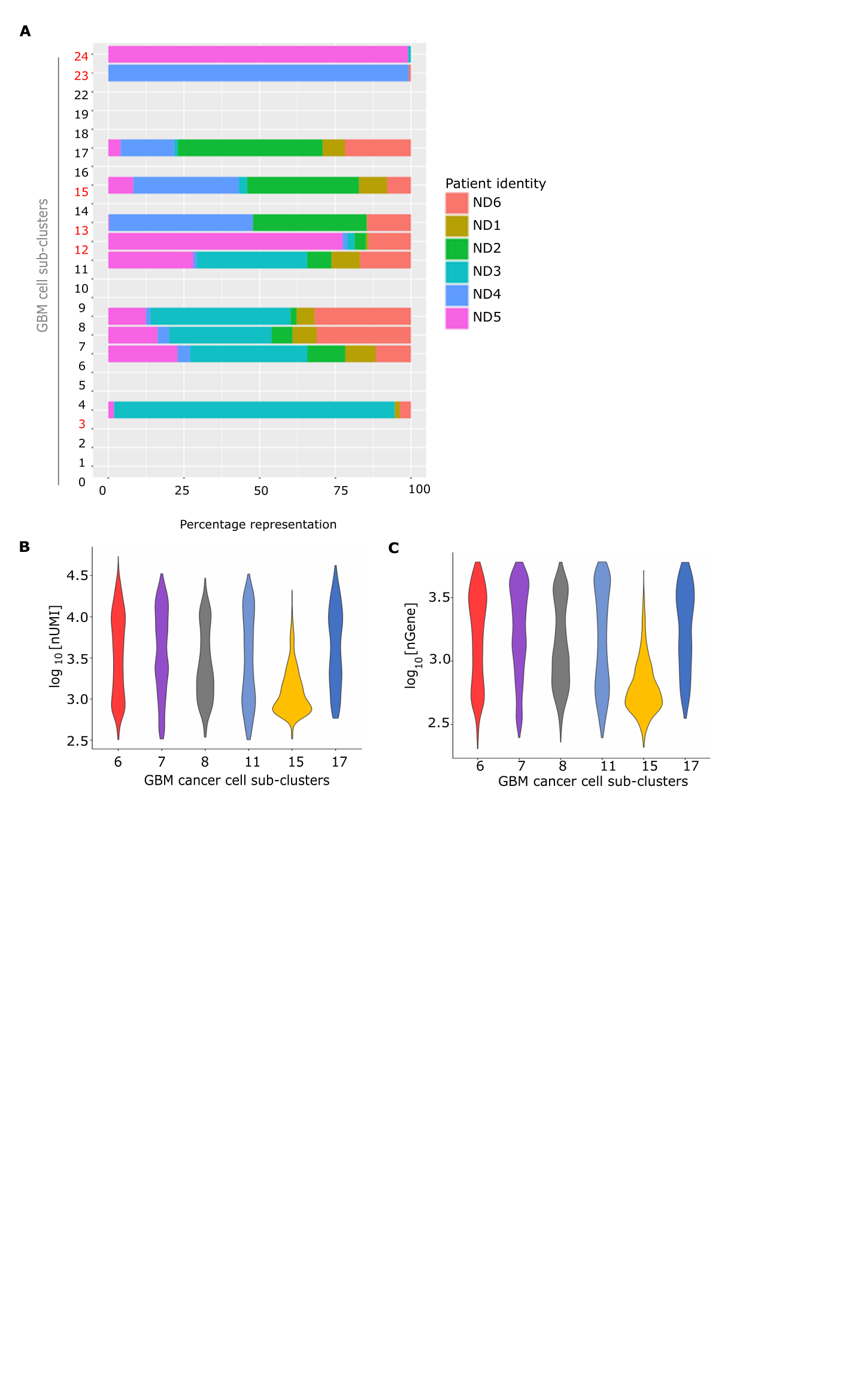

Supplement: Supplementary file 1 [file DataSheet_1.zip › Images/Supplementary Image 1.TIFF]

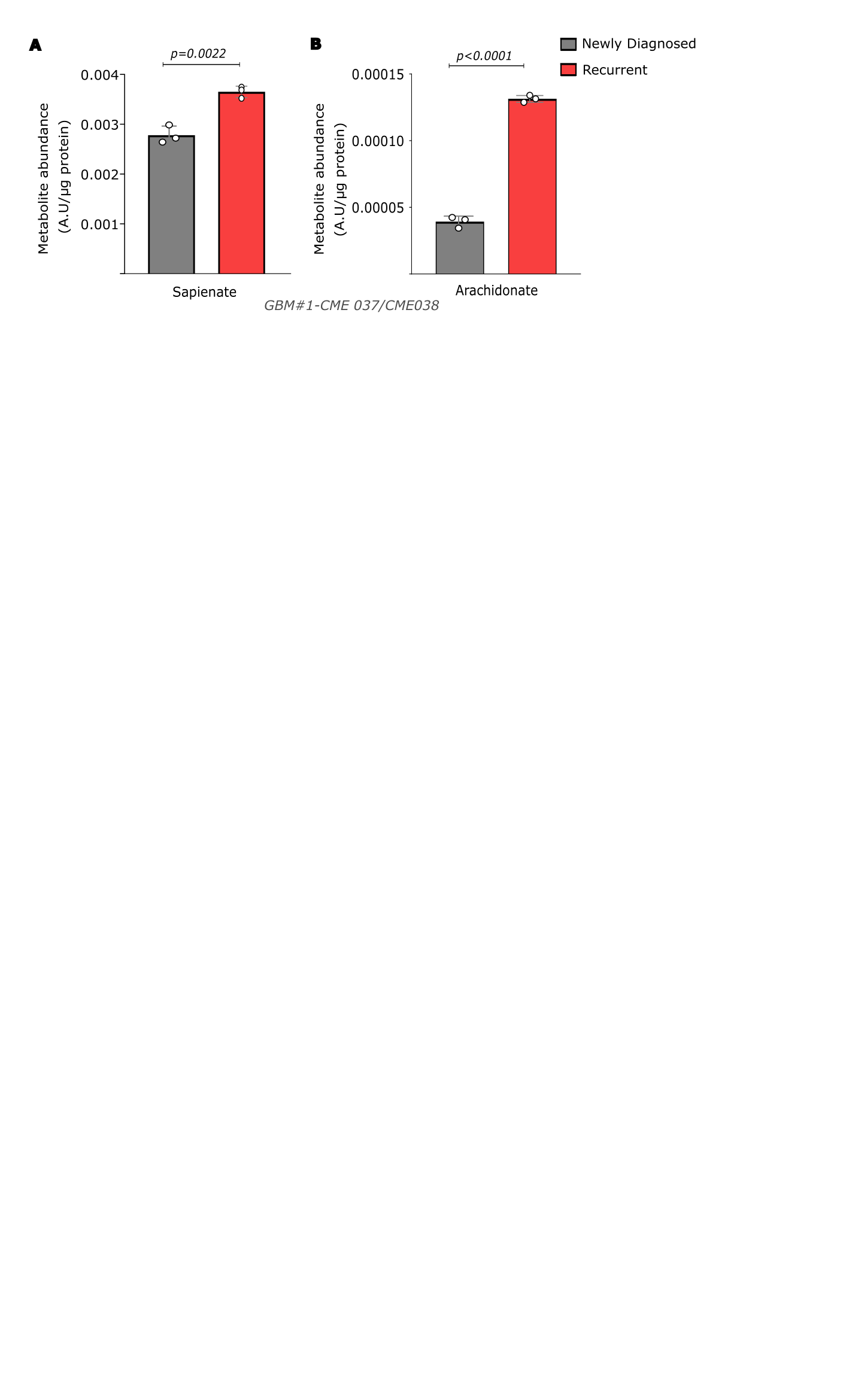

Supplement: Supplementary file 1 [file DataSheet_1.zip › Images/Supplementary Image 10.TIFF]

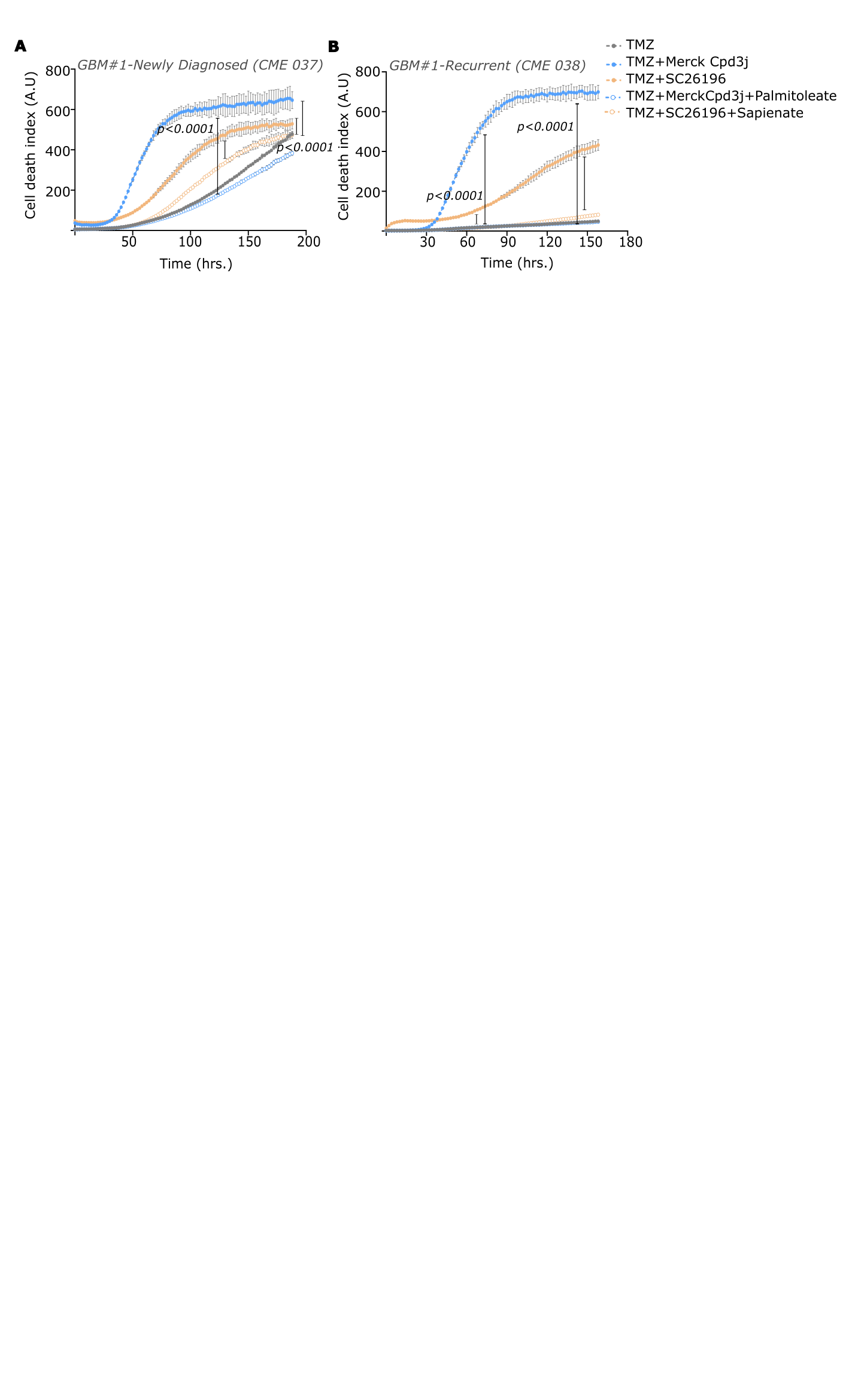

Supplement: Supplementary file 1 [file DataSheet_1.zip › Images/Supplementary Image 11.TIFF]

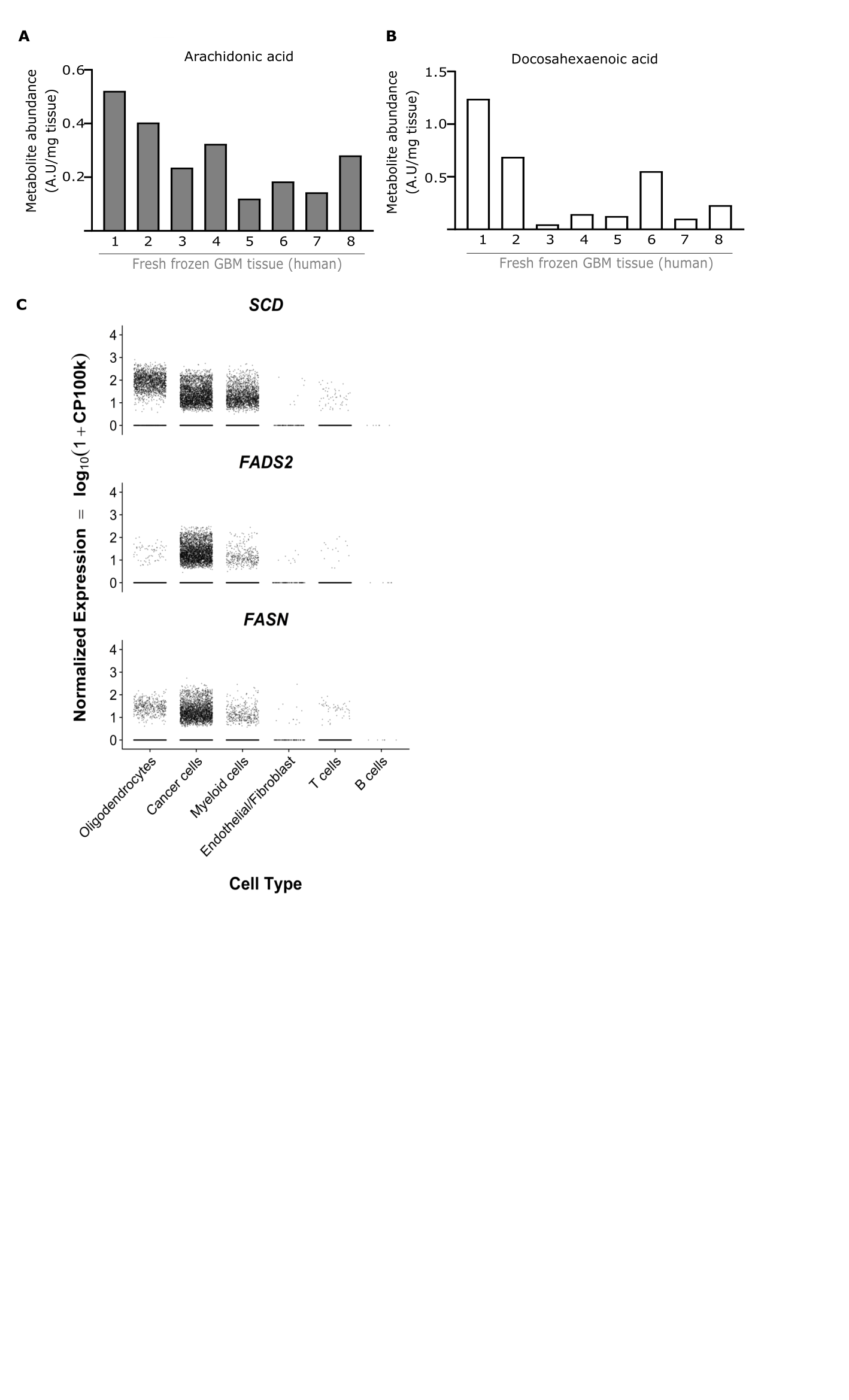

Supplement: Supplementary file 1 [file DataSheet_1.zip › Images/Supplementary Image 2.TIFF]

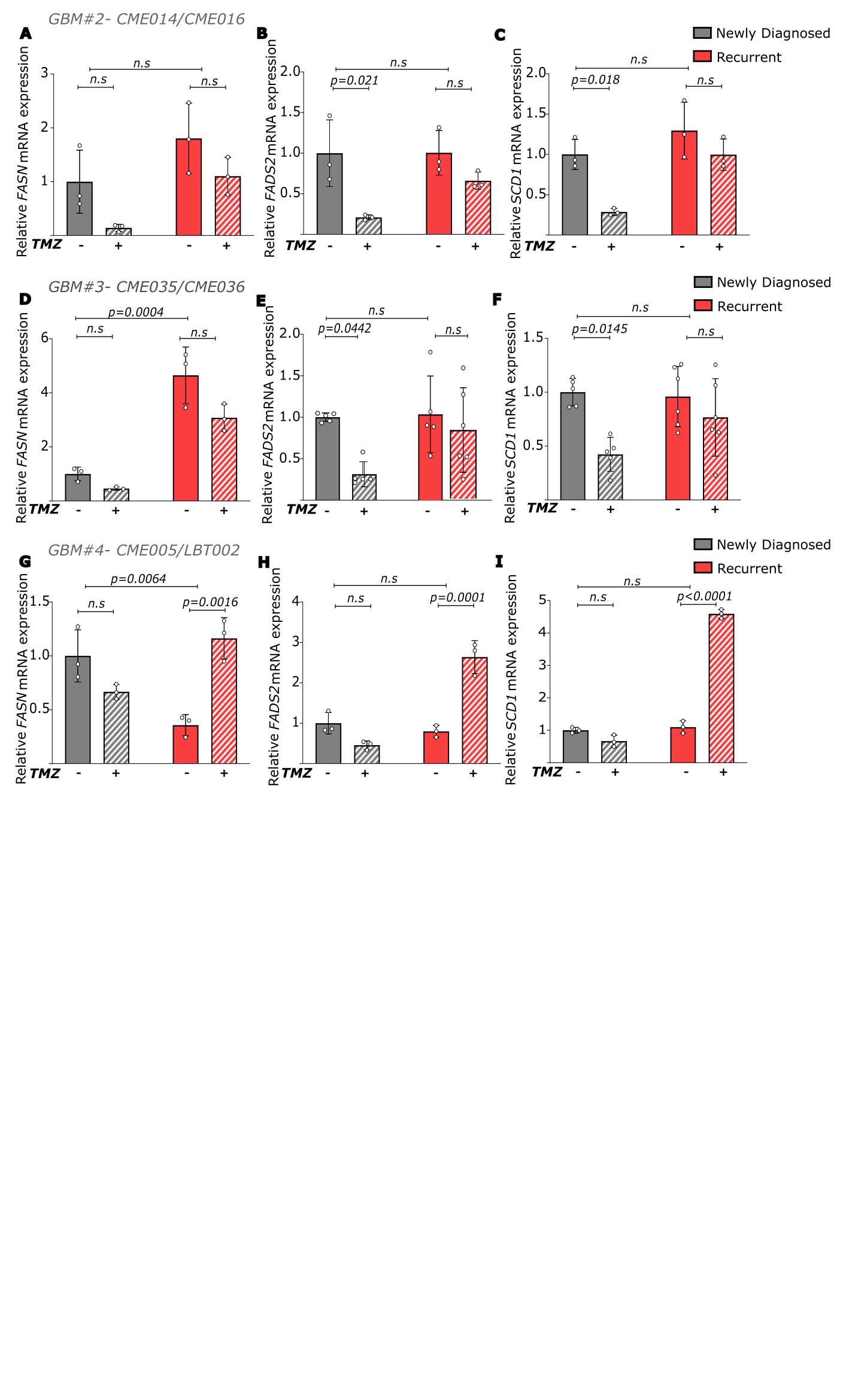

Supplement: Supplementary file 1 [file DataSheet_1.zip › Images/Supplementary Image 3.TIFF]

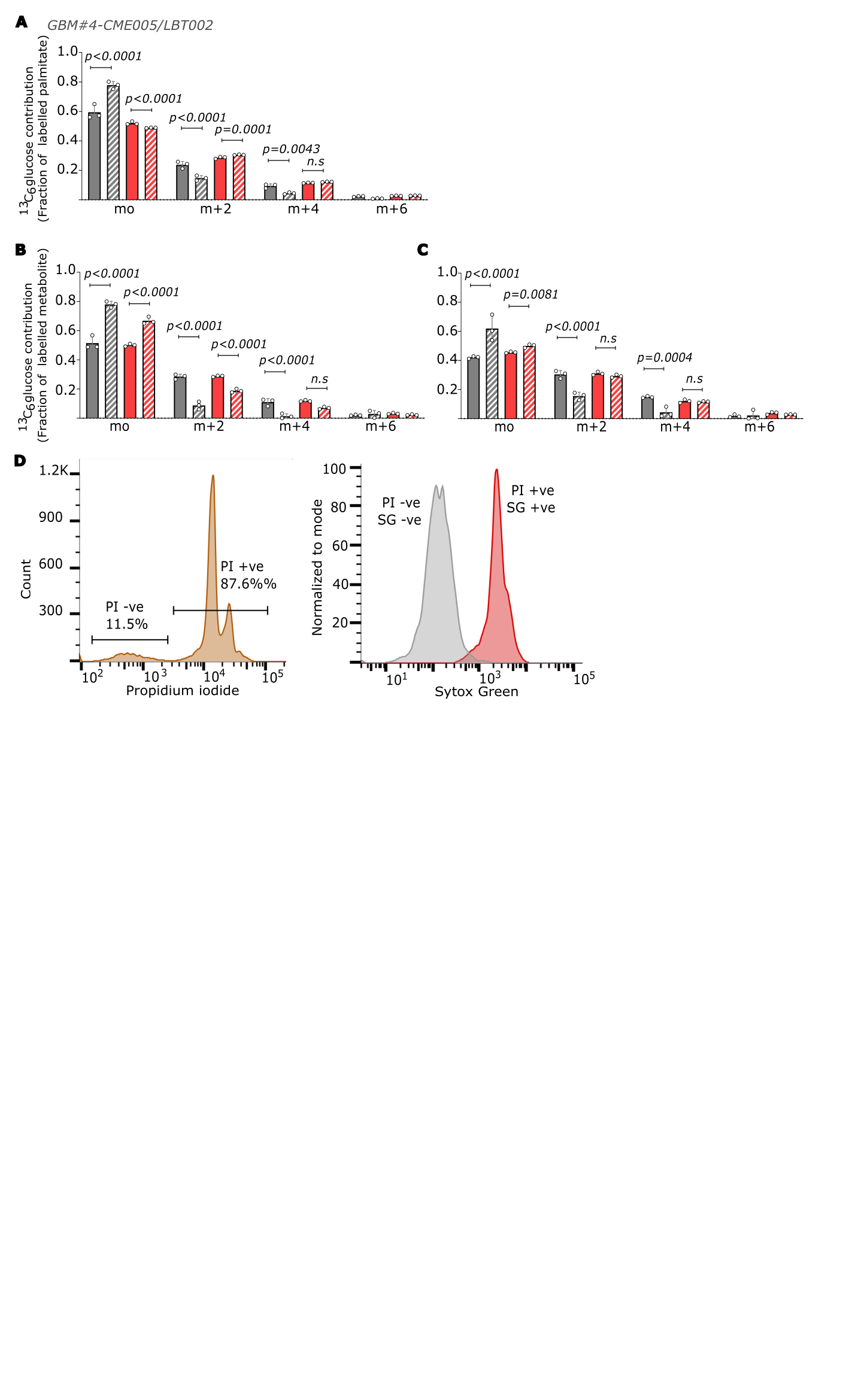

Supplement: Supplementary file 1 [file DataSheet_1.zip › Images/Supplementary Image 4.TIFF]

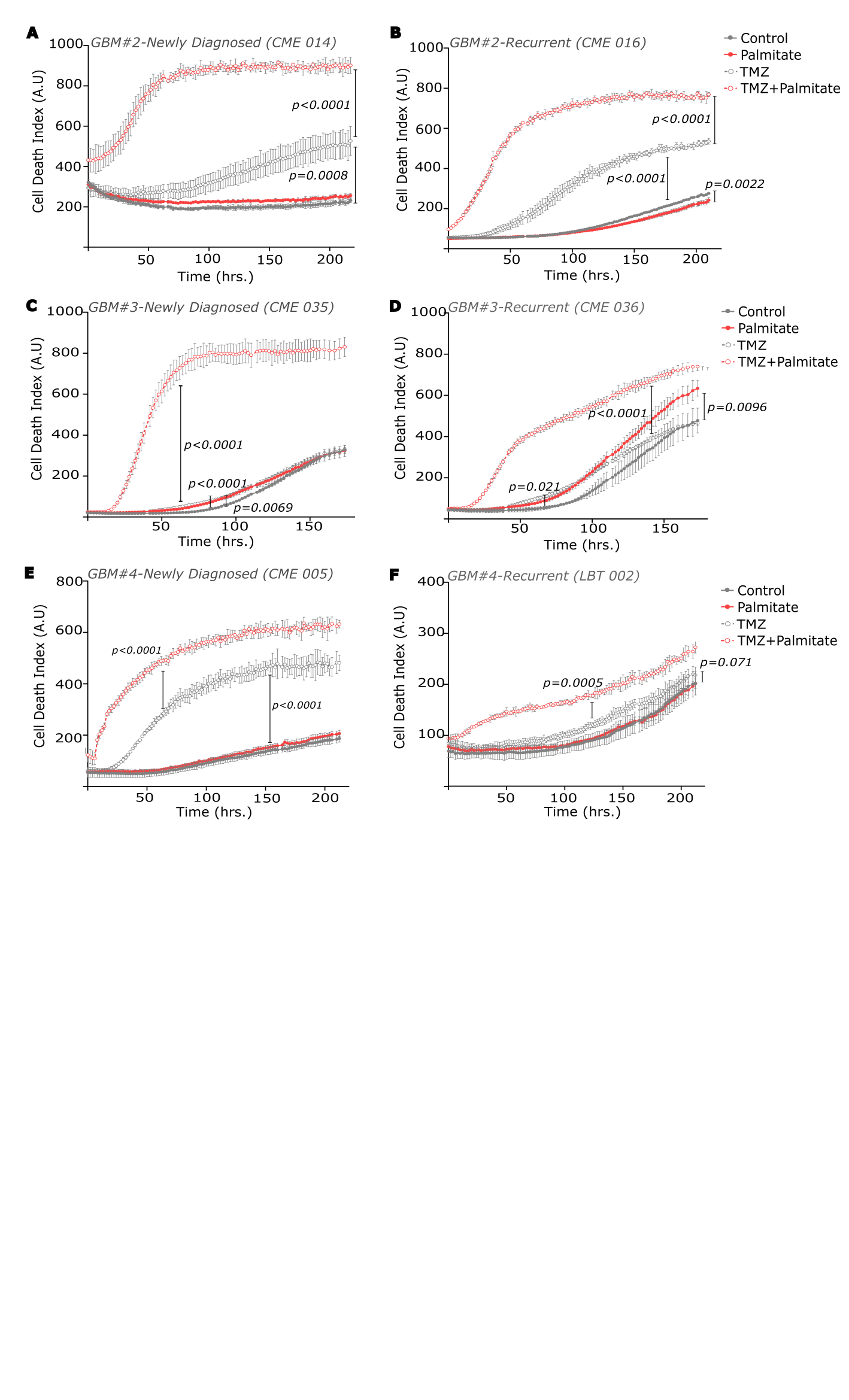

Supplement: Supplementary file 1 [file DataSheet_1.zip › Images/Supplementary Image 5.TIFF]

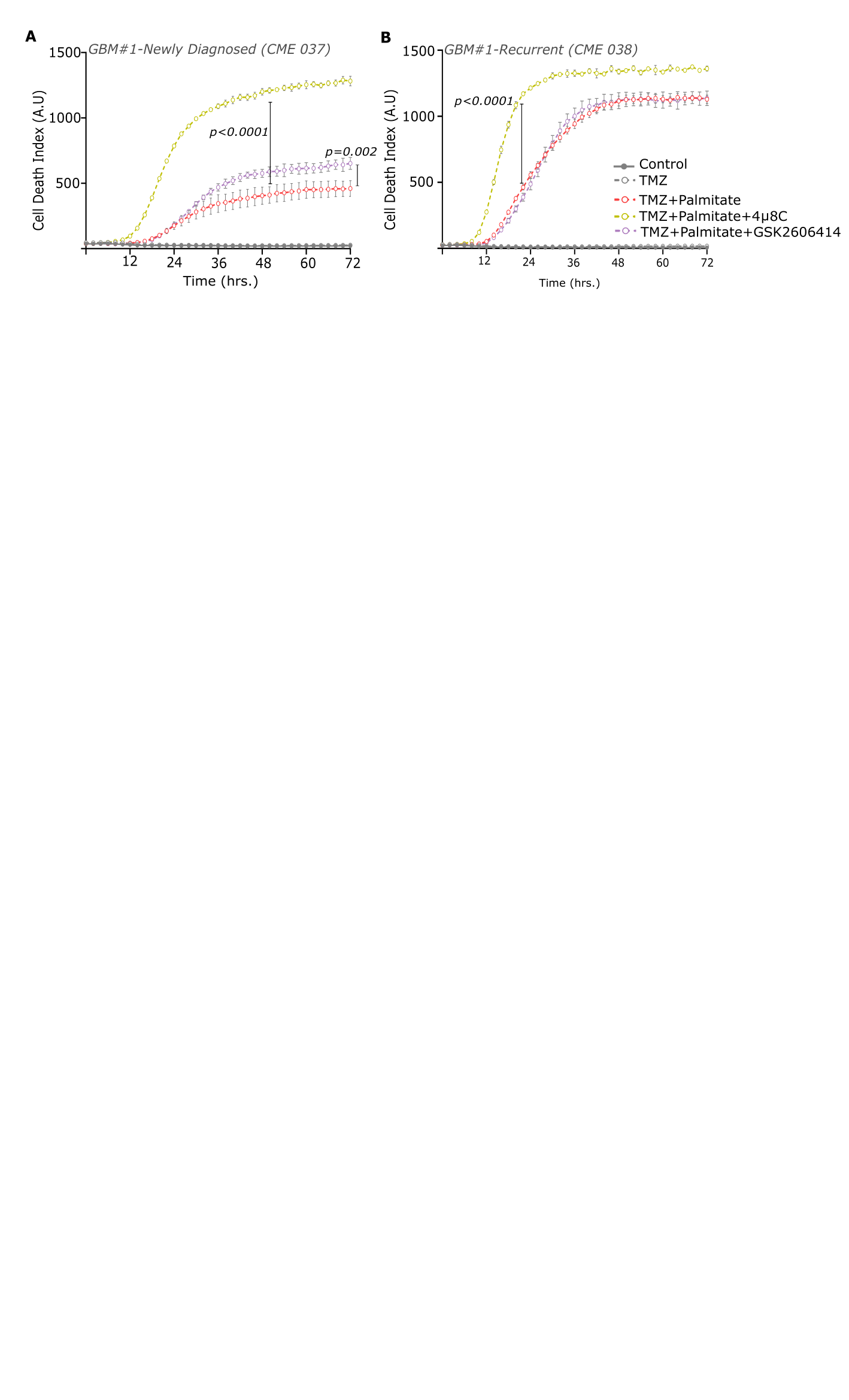

Supplement: Supplementary file 1 [file DataSheet_1.zip › Images/Supplementary Image 9.TIFF]
